# Production of IgG antibodies to pneumococcal polysaccharides is associated with expansion of ICOS^+^ circulating memory T follicular-helper cells which is impaired by HIV infection

**DOI:** 10.1371/journal.pone.0176641

**Published:** 2017-05-02

**Authors:** Laila N. Abudulai, Sonia Fernandez, Karli Corscadden, Sally A. Burrows, Michael Hunter, M. Christian Tjiam, Lea-Ann S. Kirkham, Jeffrey J. Post, Martyn A. French

**Affiliations:** 1 School of Pathology & Laboratory Medicine, The University of Western Australia, Perth, Australia; 2 Center for Vaccine and Infectious Disease Research, Telethon Kids Institute, The University of Western Australia, Perth, Australia; 3 School of Medicine & Pharmacology, The University of Western Australia, Perth, Australia; 4 Department of Infectious Diseases, Prince of Wales Hospital, Sydney, Australia; 5 Prince of Wales Clinical School, University of New South Wales, Sydney, Australia; 6 School of Paediatrics and Child Health, The University of Western Australia, Perth, Australia; 7 Department of Clinical Immunology, Royal Perth Hospital and PathWest Laboratory Medicine, Perth, Australia; Instituto Butantan, BRAZIL

## Abstract

Dysfunction of T follicular-helper (T_FH_) cells is a possible cause of impaired germinal centre (GC) and IgG antibody responses in individuals with human immunodeficiency virus-1 (HIV-1) infection and might contribute to decreased magnitude and isotype diversification of IgG antibodies to pneumococcal polysaccharides (PcPs). We examined the production of IgG1 and IgG2 antibodies to PcPs 4, 6B, 9V and 14 by enumerating antibody secreting cells (ASCs) at day (D) 7 and determining fold-increase in serum antibody levels at D28 after vaccination with unconjugated PcPs in HIV seronegative subjects (n = 20) and in HIV patients who were receiving antiretroviral therapy (ART) (n = 28) or who were ART-naive (n = 11) and determined their association with ICOS^+^ and ICOS^-^ circulating memory T_FH_ (cmT_FH_) cells (CD4^+^CD45RA^-^CD27^+^CXCR5^+^PD-1^+^) and short lived plasmablasts (SPBs) at D7, and with PcP-specific and total IgM^+^ and IgG^+^ memory B cells at D0. In HIV seronegative subjects, production of IgG1^+^ and IgG2^+^ ASCs was consistently associated with the frequency of ICOS^+^ cmT_FH_ cells but not ICOS^-^ cmT_FH_ cells or memory B cells. In contrast, post-vaccination ASCs in HIV patients, regardless of ART status, were lower than in HIV seronegative subjects and not associated with ICOS^+^ cmT_FH_ cells, the expansion of which was absent (ART-naive patients) or much lower than in HIV seronegative subjects (ART-treated patients). Production of SPBs was also lower in ART-naive patients. Fold-increase in IgG2 antibodies at D28 also correlated with ICOS^+^ cmT_FH_ cells at D7 in HIV seronegative subjects but not in HIV patients. These novel findings provide evidence that ICOS^+^ cmT_FH_ cells contribute to the regulation of PcP-specific IgG antibody responses, including isotype diversification, and that T_FH_ cell dysfunction may be a cause of impaired PcP-specific IgG antibody responses and increased susceptibility to pneumococcal disease in HIV patients.

## Introduction

Rates of invasive pneumococcal infection are increased by up to 100-fold in individuals with chronic human immunodeficiency virus-1 (HIV-1) infection and not fully corrected by antiretroviral therapy (ART) and vaccination with pneumococcal polysaccharides (PcPs) [1–3]. Decreased production of IgG antibodies against PcPs is the major cause of this problem. It has been argued that this may reflect a deficiency of IgM memory B cells and switched memory B cells caused by HIV-1 infection [4–6] because both memory B cell subpopulations contribute to IgG antibody responses against PcPs [7–9]. The IgG antibody response against PcPs is enriched for IgG2 antibodies [10, 11], which possess characteristics that are likely to enhance opsonisation of complex antigens, such as polysaccharides. Thus, IgG2 molecules are more effective than other IgG subclasses in opsonising antigens with a high epitope density [12], probably because they exhibit structural isoforms that result from differences in disulphide bonding between the hinge and Fab regions of the molecule [13–15], and can form covalent dimers at the hinge region [16, 17]. Furthermore, IgG2 antibodies, in addition to IgG1 but not IgG3 antibodies, enhance production of cytokines by dendritic cells when stimulated by antibody-opsonised lipopolysaccharide via FcγRIIa [18]. Of note, more than 60% of patients with HIV infection exhibit IgG2 deficiency [19–21].

Production of IgG2 antibodies requires isotype diversification of an IgG antibody response through class switch recombination of immunoglobulin heavy chain genes in follicular B cells, with switching to γ*2* occurring downstream of γ*3* and γ*1* [22, 23]. Immunoglobulin isotype switching in B cells is regulated by CD4^+^ T cells of the T follicular-helper (T_FH_) cell lineage during germinal centre (GC) reactions, which results in production of circulating short-lived plasmablasts (SPBs) and memory B cells [24–26]. T_FH_ cells express high levels of the chemokine receptor CXCR5, which facilitates trafficking of cells to CXCL13-rich GCs [24, 25]. Regulation of follicular B cell differentiation by T_FH_ cells is mediated by production of interleukin (IL)-21 and IL-4 and cell-surface expression of both co-stimulatory and co-inhibitory molecules of the CD28 family, particularly programmed cell death 1 (PD-1), B and T lymphocyte attenuator (BTLA) and inducible T-cell co-stimulator (ICOS) [24]. The latter molecule has a particularly important role in regulating immunoglobulin isotype switching because studies in both mice and humans have demonstrated that ICOS deficiency results in an inability to diversify immunoglobulin isotypes and impairs GC function [27–30]. Furthermore, ICOS has a key role in the development, maintenance and differentiation of GC T_FH_ cells [31, 32] and ICOS deficiency results in a decrease of CXCR5^+^CD4^+^ T cells [33]. Expression of ICOS by circulating memory (cm) T_FH_ cells has been proposed as a marker of activated T_FH_ cells [26] and ICOS^+^ cmT_FH_ cells generated after vaccination with an influenza virus vaccine are capable of inducing memory B cells to differentiate into plasma cells and their frequency correlates with the amount and affinity of influenza virus-specific antibodies [34, 35]. Unlike the other IgG subclasses [36–38], molecular regulation of IgG2 production is incompletely understood, though interferon (IFN)-γ may play a role [39, 40]. Of note, IgM memory B cells have recently been shown to enter GC reactions, produce γ*2* transcripts and differentiate into plasma cells under the influence of IFN-γ to a greater extent than IgG memory B cells [41].

The number of T_FH_ cells is increased in lymph nodes of patients with HIV-1 infection and rhesus macaques with simian immunodeficiency virus (SIV) infection [42–44]. However, their function is impaired by mechanisms that include ligation of programmed cell death ligand 1 (PD-L1) on GC B cells [45] and the suppressive effect of T follicular regulatory cells [46]. A study of IgG antibody responses following vaccination with seasonal H1N1 influenza virus in patients with HIV-1 infection provided evidence that T_FH_ ‘like’ cells (defined as CXCR5^+^CD4^+^) can be detected in the circulation as peripheral T_FH_ cells 28 days post-vaccination and that these cells promote production of IgG antibodies against influenza virus antigens [47]. However, it is unknown what role T_FH_ cells play in regulating production of IgG antibodies to PcPs, including isotype diversification, in HIV patients or, indeed, in individuals who are not infected by HIV-1.

PcPs are T-independent type 2 (TI-2) antigens which, while not requiring T cells for B cell activation, require CD4^+^ T cells for the regulation of the antibody response, as indicated by dependence on CD40/CD40L interaction [48, 49] and somatic hypermutation of antibody Fab regions, suggesting that GC reactions occur [50]. To investigate the regulation of IgG antibody responses against PcPs, including isotype diversification, by T_FH_ cells, and the effect of HIV infection on this, we examined the production of IgG1 and IgG2 antibodies in HIV seronegative subjects and HIV patients (ART-treated and -naive) following vaccination with unconjugated PcPs. We examined immunological correlates of IgG1 and IgG2 antibody production, specifically PcP-specific and total IgM^+^ and IgG^+^ memory B cells before vaccination and both ICOS^+^ and ICOS^-^ cmT_FH_ cells after vaccination. We report that production of IgG1^+^ and IgG2^+^ PcP-specific antibody secreting cells (ASCs) at day (D) 7, as well as serum IgG2 antibodies at D28, is associated with expansion of ICOS^+^ but not ICOS^-^ cmT_FH_ cells in HIV seronegative subjects but not in HIV patients.

## Results

### IgG2^+^ ASCs were more frequent than IgG1^+^ ASCs after vaccination with PcPs but both were less frequent in HIV patients

**Fig 1:**
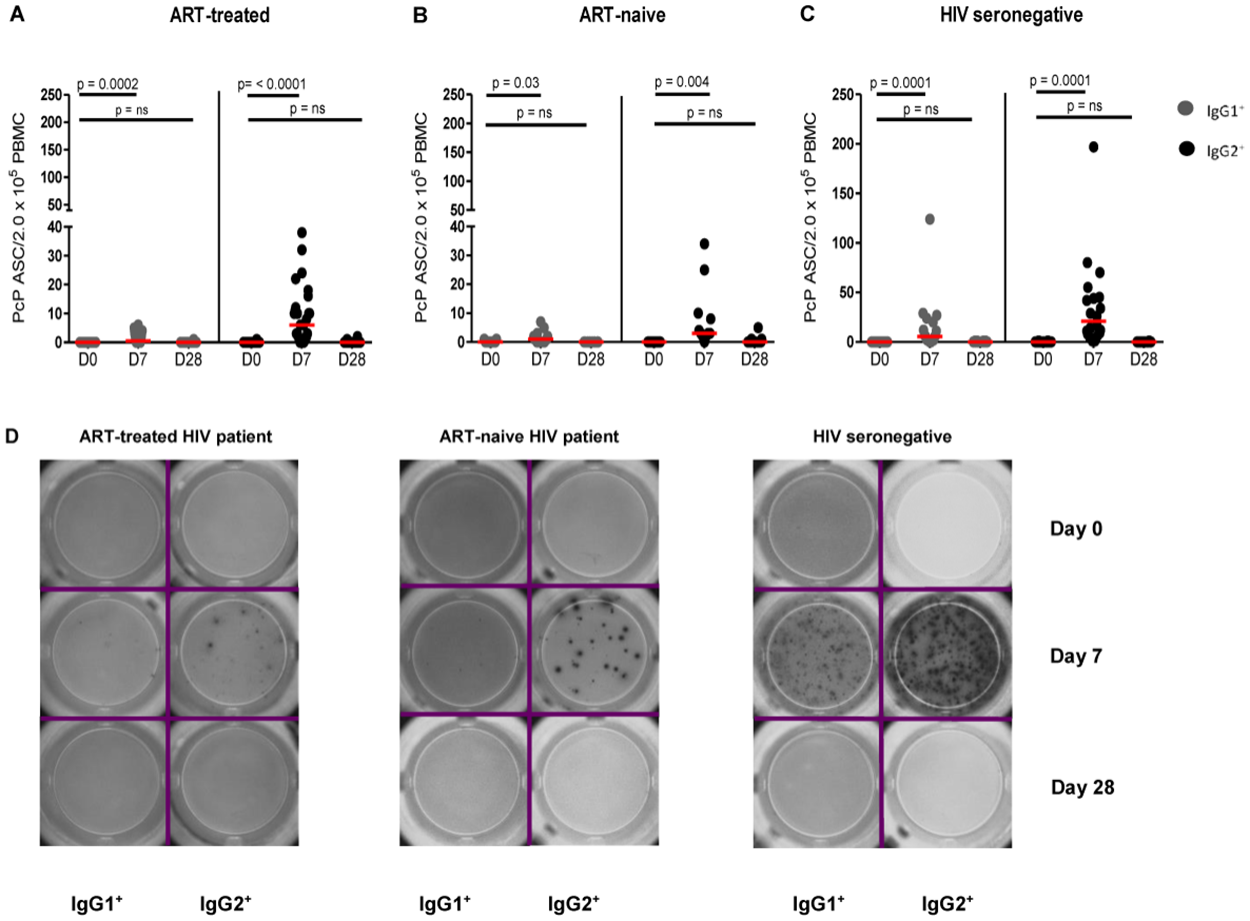
PcP serotype 4-specific IgG1^+^ and IgG2^+^ ASCs in HIV patients and HIV seronegative subjects pre- and post-vaccination with PcPs. ASCs were detected by ELISpot assays in unstimulated PBMC before (D0), one week (D7) and four weeks (D28) after vaccination with PcPs in (**A**) ART-treated HIV patients, (**B**) ART-naive HIV patients and (**C**) HIV seronegative subjects. Representative ELISpot images are shown in (**D**). Data are presented as ASC/2.0x10^5^ PBMC with background values subtracted. The horizontal lines indicate median values. Repeated measures negative binomial regression analysis and non-parametric tests for IgG1^+^ and IgG2^+^ ASC counts at D0 and D28. n.s., not significant and p<0.05 considered significant.

**Fig 2:**
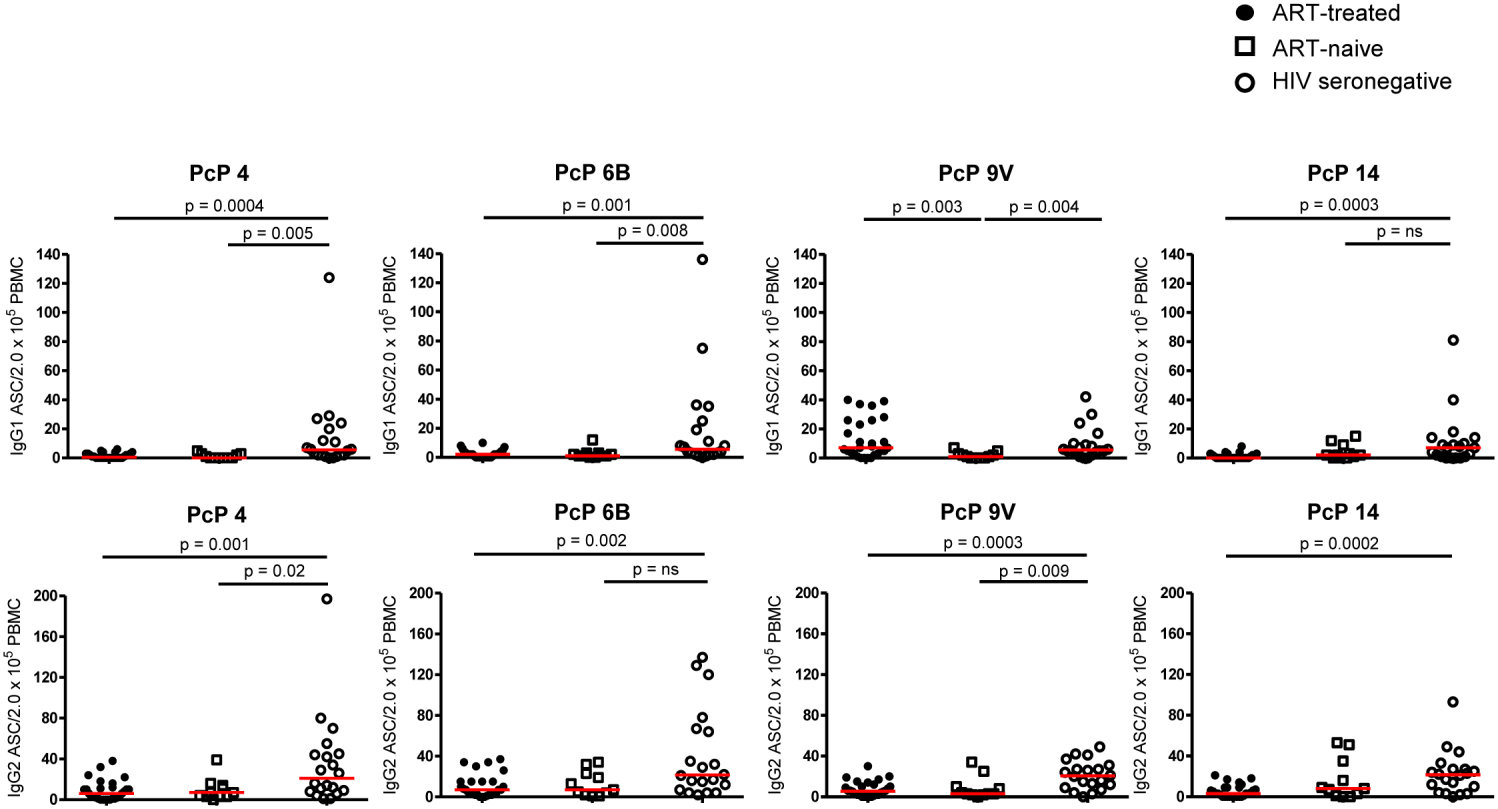
PcP-specific IgG1^+^ and IgG2^+^ ASCs in HIV patients and HIV seronegative subjects at day 7 post-vaccination with PcPs. **(A)** IgG1^+^ ASC to PcP 4, 6B, 9V and 14 **(B)** IgG2^+^ ASC to PcP 4, 6B, 9V and 14. Data are presented as ASC/2x10^5^ PBMC. The horizontal lines indicate median values. Median values of IgG1^+^ ASC/2 x10^5^ PBMC in ART-treated, ART-naive and HIV seronegative subjects to PcP 4: 0.5, 0 and 6, respectively; PcP 6B: 2, 1 and 6, respectively; PcP 9V: 1, 1 and 6, respectively and PcP 14: 0, 2 and 7, respectively. Median values of IgG2^+^ ASC/ 2x10^5^ PBMC in ART-treated, ART-naive and HIV seronegative subjects to PcP 4: 6, 7 and 21, respectively; PcP 6B: 7, 7 and 22, respectively; PcP 9V: 6, 3 and 21, respectively and PcP 14: 3, 8 and 22, respectively. Differences between groups were tested using Mann-Whitney tests. n.s., not significant and p<0.05 considered significant.

After vaccination with unconjugated PcPs, IgG1^+^ and IgG2^+^ ASCs for PcP serotypes 4 (Fig 1A–1D), 6B, 9V and 14 (S1A–S1I Fig), were detected at D7 but not D28. As expected [51], IgG2^+^ ASCs were more frequent than IgG1^+^ ASCs. For all four PcP serotypes, IgG1^+^ and IgG2^+^ ASCs were more frequent in HIV seronegative subjects than ART-treated and ART-naive HIV patients at D7 after vaccination (Fig 2A and 2B).

### Expansion of plasmablasts was maximal 7 days after vaccination with PcPs and impaired in ART-naive HIV patients

**Fig 3:**
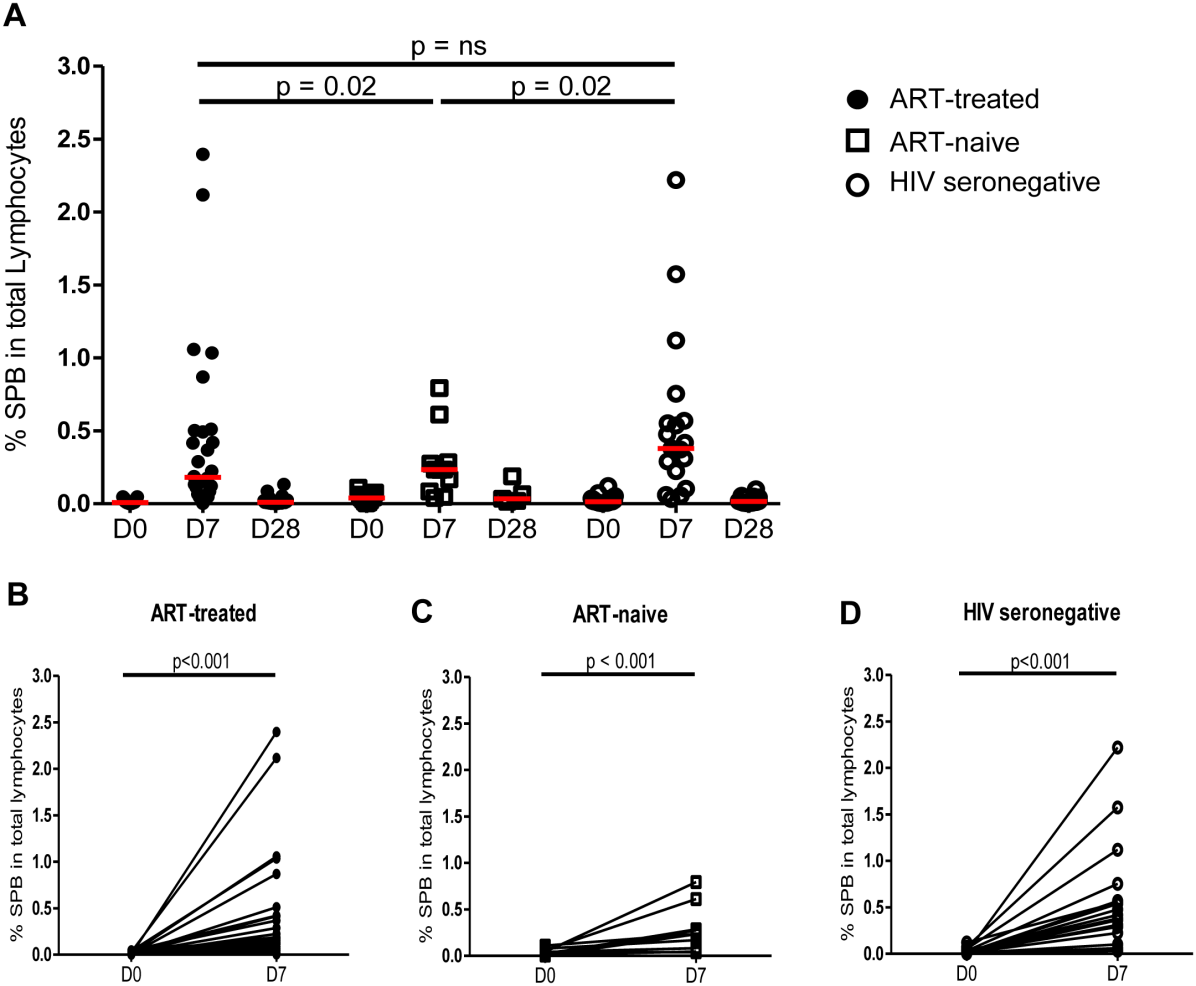
Circulating SPB in HIV patients and HIV seronegative subjects at day 7 post-vaccination with PcPs. (**A**) SPB (CD20^-^CD27^++^CD38^++^) at D0, D7 and D28 in the 3 study groups. The increase in SPB between D0 and D7 is shown for (**B**) ART-treated patients (**C**) ART-naive patients and (**D**) HIV seronegative subjects. Data were analysed using linear mixed models. n.s., not significant and p<0.05 considered significant.

We also examined the effect of vaccination with PcPs on production of circulating SPBs, which were detected using the gating strategy shown in S2 Fig. As demonstrated for ASCs, the proportion of lymphocytes with characteristics of SPBs (CD20^-^CD27^++^CD38^++^) was higher than baseline at D7 post-vaccination and returned to pre-vaccination values at D28 (Fig 3A). While all study groups demonstrated an increase of SPBs from day 0 to day 7 (p<0.001; Fig 3B–3D), ART-naive HIV patients exhibited lower responses than HIV seronegative subjects and ART-treated HIV patients (p<0.02 for both; Fig 3A), who did not differ from each other.

### Proportions of ICOS^+^ cmT_FH_ cells correlated with IgG1^+^ and IgG2^+^ ASCs after vaccination with PcPs but not in HIV patients

**Fig 4:**
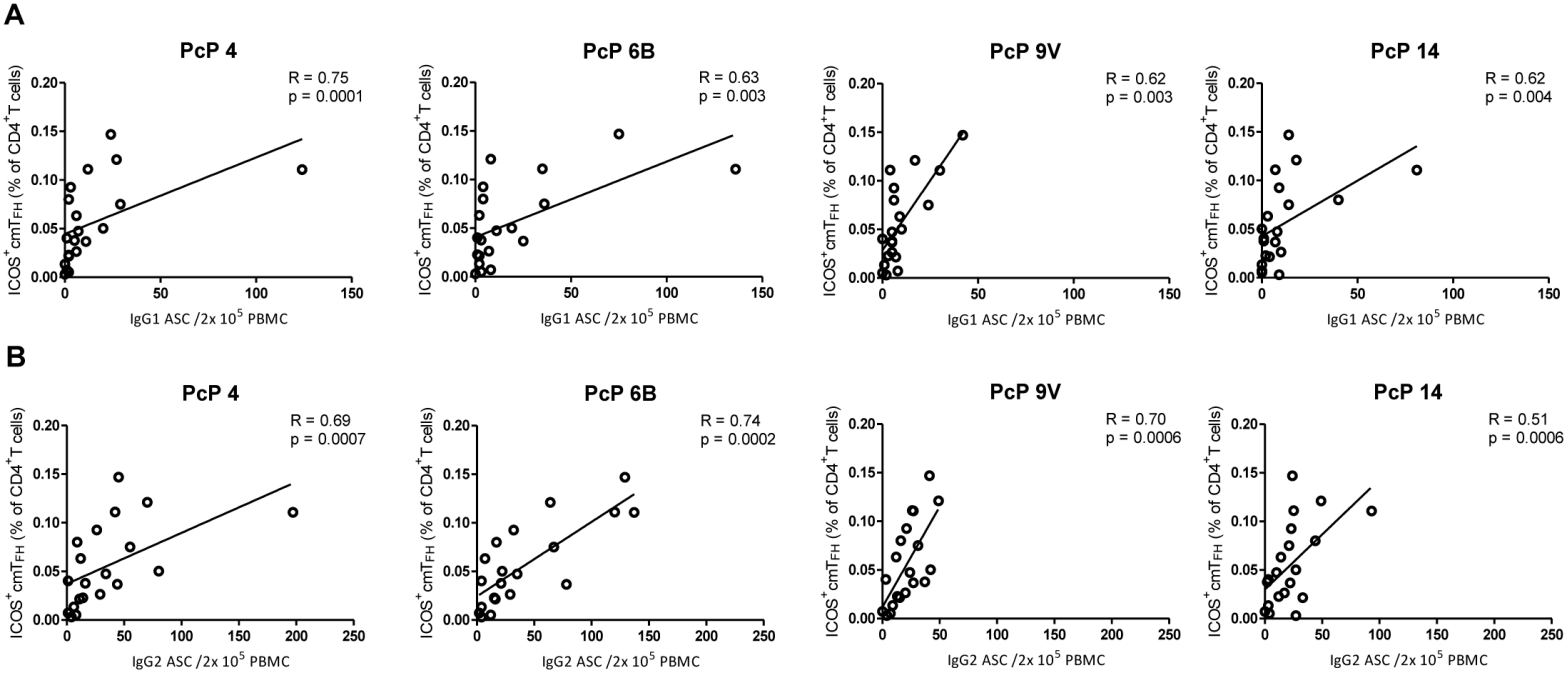
PcP-specific IgG1^+^ and IgG2^+^ ASCs correlated with ICOS^+^ cmT_FH_ cells in HIV seronegative subjects at D7 post-vaccination. **(A)** IgG1^+^ ASC **(B)** IgG2^+^ ASC. Data were analysed using Spearman’s rank correlation test.

If cmT_FH_ cells contribute to the regulation of IgG1^+^ and IgG2^+^ ASC production after vaccination with PcPs, it is likely that cmT_FH_ cells will increase at the same time as the ASCs. We therefore examined the correlation of IgG1^+^ and IgG2^+^ ASCs at D7 with two cmT_FH_ cell populations, characterised as CD4^+^ T cells with a central memory phenotype (CD27^+^CD45RA^-^) expressing CXCR5 and PD-1 with or without ICOS, according to the gating strategy illustrated in S3 Fig. The cmT_FH_ cells were predominantly central memory T cells (median frequency was 88%, 87% and 79% in HIV seronegative subjects, ART-treated and ART-naive HIV patients, respectively; data not shown). In contrast, the median frequency of cmT_FH_ cells with an effector memory phenotype was less than 4% in HIV patients and HIV seronegative subjects (data not shown). In HIV seronegative subjects, production of IgG1^+^ and IgG2^+^ ASCs for all four PcP serotypes positively correlated with the proportion of ICOS^+^ cmT_FH_ cells at D7 after vaccination (Fig 4A and 4B). In contrast, neither IgG1^+^ nor IgG2^+^ ASCs correlated with ICOS^-^ cmT_FH_ cells in HIV seronegative subjects (R≤0.36, p≥0.12 and R≤0.35, p≥0.13, respectively, S1 Table). In contrast to HIV seronegative subjects, IgG1^+^ and IgG2^+^ ASCs did not correlate with proportions of ICOS^+^ cells in ART-treated (R = -[0.01–0.06], p≥0.09 and R = -[0.01–0.20], p≥0.31, respectively, S2 Table) or ART-naive HIV patients (R≤0.41, p≥0.21 and R≤0.36, p≥0.27, respectively, S3 Table). In addition, no correlations between IgG1^+^ and IgG2^+^ ASC and ICOS^-^ cmT_FH_ cells were observed in ART-treated HIV patients (R = -[0.12–0.11], p≥0.37 and R = -[0.03–0.15], p≥0.45, respectively, S2 Table) or ART-naive HIV patients (R≤9.3x10^4^, p≥0.29 and R≤0.32, p≥0.33, respectively S3 Table). Based on these findings, we concluded that the ICOS^+^ subpopulation of cmT_FH_ cells was most strongly associated with IgG antibody responses to all four PcP serotypes after vaccination.

### ICOS^+^ cmT_FH_ cells were increased in ART-naive patients whereas ICOS^-^ cmT_FH_ cells were decreased in ART-treated patients

**Fig 5:**
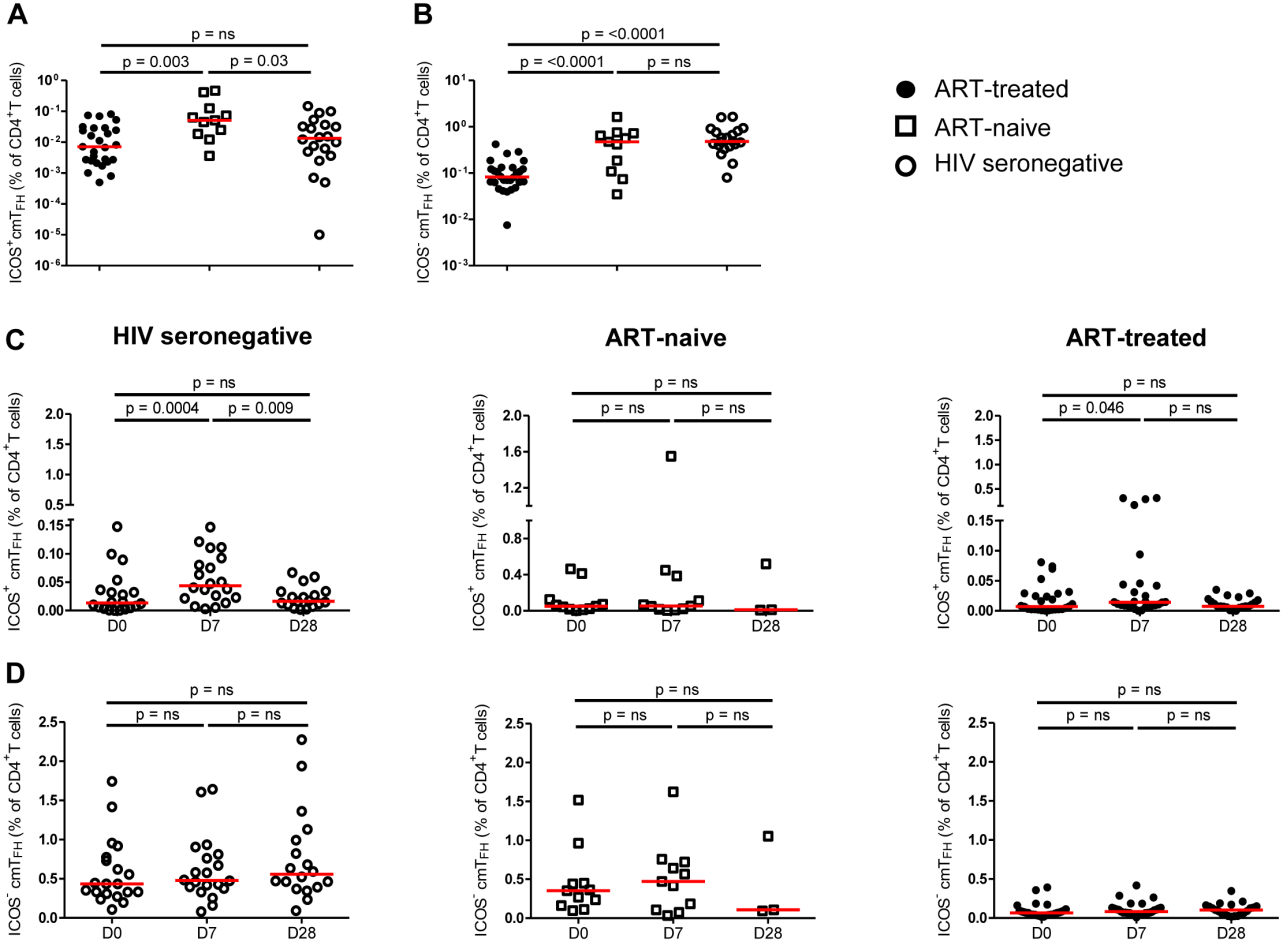
Proportions of ICOS^+^ and ICOS^-^ cmT_FH_ cells pre- and post- vaccination in HIV patients and HIV seronegative subjects. **(A)** ICOS^+^ cmT_FH_ cells, **(B)** ICOS^-^ cmT_FH_ cells. Data are presented on a log scale and were analysed by Mann-Whitney tests. n.s., not significant and p<0.05 considered significant, **(C)** Following vaccination with PcPs, ICOS^+^ cmT_FH_ cells increased at D7, compared with D0, in HIV seronegative subjects but not in ART-naive HIV patients and to a lesser and more variable degree in ART-treated patients and **(D**) ICOS^-^ cmT_FH_ cells did not increase in any of the study groups. Data were analysed by Wilcoxon signed-rank test and Kruskal Wallis test. n.s., not significant and p<0.05 considered significant.

To determine if cmT_FH_ cells were increased, as reported for lymph node T_FH_ cells in HIV-1 and SIV infection [42, 44, 45], we examined the frequency of ICOS^+^ and ICOS^-^ cmT_FH_ cells at D0 in HIV patients and HIV seronegative subjects. Proportions of ICOS^+^ cmT_FH_ cells were higher in ART-naive patients compared to ART-treated patients (p = 0.003) and HIV seronegative subjects (p = 0.03), who did not differ from each other (Fig 5A). In contrast, proportions of ICOS^-^ cmT_FH_ cells were lower in ART-treated patients compared to both ART-naive patients and HIV seronegative subjects (p<0.0001), who did not differ from each other (Fig 5B).

### ICOS^+^ cmT_FH_ cells expanded after vaccination with PcPs but not in HIV patients

At D7 post-vaccination, proportions of ICOS^+^ cmT_FH_ cells were higher, compared with day 0, in HIV seronegative subjects (p = 0.0004; Fig 5C). ART-treated HIV patients also exhibited higher proportions of ICOS^+^ cmT_FH_ cells at D7 compared with D0 (p = 0.046, Fig 5C), but this was more variable and of lower magnitude than in HIV seronegative subjects. The median value of ICOS^+^ cmT_FH_ cells increased by only 0.007% in ART-treated patients compared with 0.03% in HIV seronegative subjects. In contrast, proportions of ICOS^+^ cmT_FH_ cells did not increase from D0 to D7 in ART-naive HIV patients (Fig 5C). In HIV seronegative subjects, proportions of ICOS^+^ cmT_FH_ cells at D28 were lower than D7 (p = 0.009) and did not differ from D0 (Fig 5C). No differences in ICOS^-^ cmT_FH_ cells were observed over the vaccination period in HIV patients and HIV seronegative subjects (Fig 5D), though D28 data were available for only three ART-naive patients.

### Frequencies of ICOS^+^ cmT_FH_ cells after vaccination, but not memory B cells before vaccination, exhibited a consistent relationship with PcP-specific ASCs after vaccination but not in HIV patients

**Fig 6:**
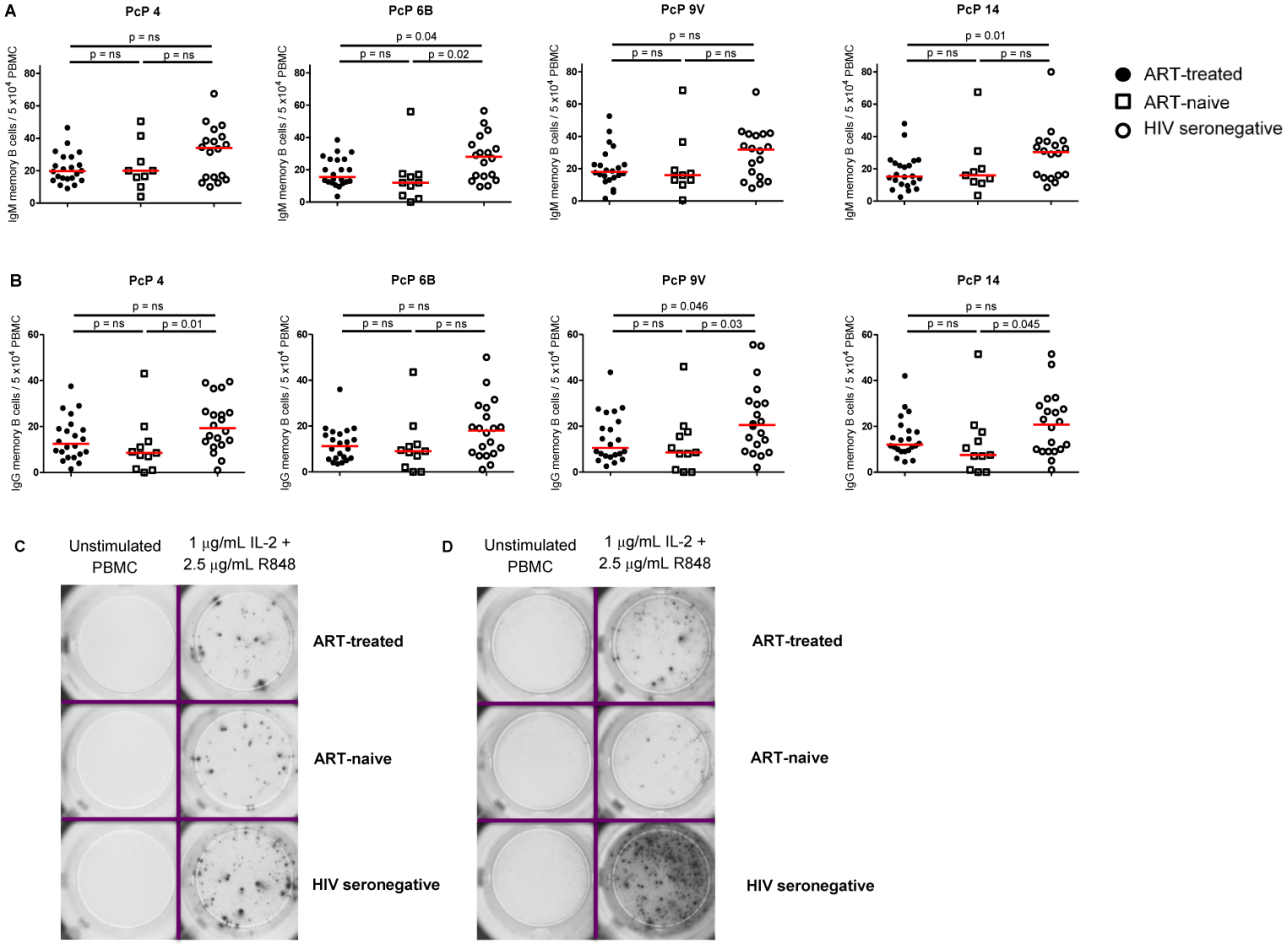
PcP specific IgG^+^ and IgM^+^ memory B cells in HIV patients and HIV seronegative subjects pre-vaccination with PcPs. PBMCs were stimulated *in vitro* for 5 days with a combination of IL-2 and R848. (**A**) IgM^+^ memory B cells to PcP 4, 6B, 9V and 14 (**B**) IgG^+^ memory B cells to PcP 4, 6B, 9V and 14. Representative ELISpot images for PcP serotype 4-specific (**C**) IgM^+^ memory B cells and (**D**) IgG^+^ memory B cells. Data are presented as memory B cells /5.0x10^4^ PBMC with background values subtracted. The horizontal lines indicate median values. Differences between groups were tested using Mann-Whitney tests. n.s., not significant and p<0.05 considered significant.

Production of PcP-specific ASCs after vaccination is potentially affected by several variables, including the numbers and/or function of memory B cells and T_FH_ cells, both of which are adversely affected by HIV infection [4–6, 42, 44, 45]. We therefore examined the relationship of IgG1^+^ and IgG2^+^ PcP-specific ASCs at D7 after vaccination with the frequencies of ICOS^+^ and ICOS^-^ cmT_FH_ cells at that time, and with the frequencies of total and PcP-specific IgG^+^ and IgM^+^ memory B cells before vaccination, in HIV patients and HIV seronegative subjects. As expected [4–8], before vaccination IgM^+^ PcP-specific memory B cells were more abundant than IgG^+^ PcP-specific memory B cells and the frequency of both IgM^+^ and IgG^+^ PcP-specific memory B cells was lower in HIV patients than HIV seronegative subjects (Fig 6A–6D), although some differences were not statistically significant. Moreover, no differences in PcP-specific IgG^+^ and IgM^+^ memory B cells were observed between HIV patient groups (Fig 6A and 6B).

In contrast to the frequencies of total and PcP-specific IgG^+^ and IgM^+^ memory B cells before vaccination, the frequencies of ICOS^+^ cmT_FH_ cells at D7 after vaccination exhibited by far the most consistent relationship with frequencies of ASCs producing antibodies to all four serotypes of PcPs at D7 after vaccination (Table 1 and S4–S6 Tables). Furthermore, the relationships between ICOS^+^ cmT_FH_ cells and PcP-specific ASCs observed in HIV seronegative subjects were larger than those observed in ART-treated HIV patients for IgG1^+^ ASCs for all 4 serotypes (p≤0.005) and for IgG2^+^ ASCs, though less so for 9V and 14 (PcP 4, p = 0.003; PcP 6B, p<0.001; PcP 9V, p = 0.09; PcP 14, p = 0.09). These relationships were also larger when HIV seronegative subjects were compared to ART-naive HIV patients for IgG1^+^ ASCs for all 4 serotypes (p≤0.001) and for IgG2^+^ ASCs, apart from PcP 9V (PcP 4, p = 0.002; PcP 6B, p<0.001; PcP 9V, p = 0.15; PcP 14, p = 0.045). The relationship between ICOS^+^ cmT_FH_ cells and IgG1^+^ or IgG2^+^ ASCs in ART-treated HIV patients was not different to that in ART-naive patients (Table 1 and S4–S6 Tables).

**Table 1:** Immune correlates of PcP 4-specific IgG1^+^ and IgG2^+^ ASCs after vaccination with PcPs in HIV patients and HIV seronegative subjects.

Immune correlate	Interaction p-value						Incident rate ratio (IRR) (95% confidence interval)					
	ART-treated v HIV seronegative		ART-naive v HIV seronegative		ART-treated v ART-naive		ART-treated HIV patients		ART-naive HIV patients		HIV seronegative subjects	
	IgG1^+^ ASCs	IgG2^+^ ASCs	IgG1^+^ ASCs	IgG2^+^ ASCs	IgG1^+^ ASCs	IgG2^+^ ASCs	IgG1^+^ ASCs	IgG2^+^ ASCs	IgG1^+^ ASCs	IgG2^+^ ASCs	IgG1^+^ ASCs	IgG2^+^ ASCs
CD4^+^ T cell count (D0), cells/μL	0.24	0.09	0.64	0.76	0.88	0.50	0.99 (0.99,1.00) p = 0.84	0.99 (0.99,1.00) p = 0.21	1.00 (0.99,1.00) p = 0.93	1.00 (0.99,1.00) p = 0.81	1.00 (0.99, 1.00) p = 0.16	1.00 (0.99, 1.00) p = 0.24
Total IgM memory B cells (CD20^+^CD27^+^IgM^+^) (D0), %	0.65	0.17	0.20	0.40	0.24	0.76	0.96 (0.85,1.09) p = 0.65	0.93 (0.83 1.04) p = 0.18	0.64 (0.33,1.25) p = 0.19	0.88 (0.64,1.21) p = 0.44	0.99 (0.92, 1.09) p = 0.98	1.01 (0.95, 1.08) p = 0.67
Total IgG memory B cells (CD20^+^CD27^+^IgG^+^) (D0), %	0.32	0.92	0.39	0.61	0.79	0.60	0.94 (0.81,1.10) p = 0.45	1.03 (0.92,1.16) p = 0.57	0.89 (0.60,1.32) p = 0.56	0.96 (0.73,1.25) p = 0.75	1.09 (0.86, 1.38) p = 0.48	1.05 (0.84, 1.31) p = 0.69
PcP 4-specific IgM^+^ memory B cells (D0), counts	0.93	0.68	0.64	0.31	0.78	0.63	0.99 (0.90,1.09) p = 0.85	0.99 (0.93,1.04) p = 0.64	1.00 (0.94,1.08) p = 0.83	1.00 (0.96,1.06) p = 0.84	0.99 (0.93, 1.04) p = 0.64	0.97 (0.93, 1.01) p = 0.19
PcP 4-specific IgG^+^ memory B cells (D0), counts	0.92	0.85	0.75	0.30	0.71	0.27	1.00 (0.92,1.09) p = 0.99	0.98 (0.92,1.04) p = 0.44	1.02 (0.95,1.10) p = 0.57	1.03 (0.96,1.10) p = 0.43	1.01 (0.95, 1.07) p = 0.85	0.98 (0.94, 1.03) p = 0.49
ICOS^+^ cmT_FH_ cells (D7), %	<0.001	0.003	<0.001	0.002	0.12	0.79	5.2x10^-4^ (9.19x10^-8^, 2.99) p = 0.09	0.30 (2.64x10^-3^, 33.73) p = 0.62	0.67 (0.06, 7.83) p = 0.75	0.59 (0.10, 3.42) p = 0.56	3.01x10^12^ (7.8x10^6^, 1.17x10^18^) p = <0.001	2.22x10^8^ (764.18, 6.48x10^13^) p = 0.003
ICOS^-^ cmT_FH_ cells (D7), %	0.14	0.61	0.99	0.68	0.14	0.73	3.8x10^-3^ (6.25x10^-7^, 23.17) p = 0.21	0.58 (2.22x10^-3^, 151.50) p = 0.85	3.23 (0.32, 32.33) p = 0.32	1.61 (0.31, 8.27) p = 0.57	3.24 (0.41, 25.48) p = 0.26	2.59 (0.57, 11.66) p = 0.22

We also examined the ‘strength’ of the relationship of PcP-specific ASCs after vaccination with the frequencies of ICOS^+^ cmT_FH_ cells after vaccination and both total and PcP-specific IgG^+^ and IgM^+^ memory B cells before vaccination, within each of the three study groups by calculating incident rate ratios (IRRs) (Table 1 and S4–S6 Tables). The IRRs for these relationships were very large (Table 1 and S4–S6 Tables) due to the small range of values of ICOS^+^ cmT_FH_ cells, therefore their interpretation is more relevant in terms of a change in the ICOS^+^ cmT_FH_ variable of 0.01, where the IRR is raised to the power of 0.01. In HIV seronegative subjects, an increase in 0.01 units of ICOS^+^ cmT_FH_ cells was associated with a 33%, 28%, 19% and 25% increase in IgG1^+^ ASCs for PcP 4, 6B, 9V and 14, respectively (p≤0.002 for all serotypes) and a 21%, 22%, 9% and 13% increase in IgG2^+^ ASC for PcP 4 (p = 0.003), 6B (p<0.001), 9V (p = 0.15) and 14 (p = 0.048), respectively (Table 1 and S4–S6 Tables). In contrast, in ART-treated HIV patients, an increase in 0.01 units of ICOS^+^ cmT_FH_ cells was associated with a 7%, 1%, 6% and 0% decrease in IgG1^+^ ASCs (p≥0.04) and a 1%, 2%, 2% and 1% decrease in IgG2^+^ ASC (p≥0.26) for PcP 4, 6B, 9V and 14, respectively (Table 1 and S4–S6 Tables). A similar trend was observed in ART-naive HIV patients, where a change in 0.01 units of ICOS^+^ cmT_FH_ cells was associated with a 1%, 1%, 0% and 2% decrease in IgG1^+^ ASCs (p≥0.27) and a 1%, 1%, 2% and 1% decrease in IgG2^+^ ASCs (p≥0.35) for PcP 4, 6B, 9V and 14, respectively (Table 1 and S4–S6 Tables). No associations between ICOS^-^ cmT_FH_ cells and IgG1^+^ or IgG2^+^ ASCs after vaccination were observed in HIV patients and HIV seronegative subjects.

### The fold-increase in serum PcP-specific IgG2 antibodies at D28 after vaccination correlated with proportions of ICOS^+^ cmT_FH_ cells at D7 but not in HIV patients

Having shown that ICOS^+^ cmT_FH_ cells were the strongest correlate with markers of an early IgG antibody response to PcPs (IgG1^+^ and IgG2^+^ ASCs) in HIV seronegative subjects, but not HIV patients, we sought to determine if the fold-increase in serum PcP-specific IgG1 and IgG2 antibody levels at D28 following vaccination was also associated with ICOS^+^ cmT_FH_ cells at D7. Over 80% of HIV seronegative subjects and HIV patients exhibited a >2-fold increase [52] in IgG1 and IgG2 antibodies to the 4 PcPs, but as expected [53] there were few differences between groups (S4A and S4B Fig). In HIV seronegative subjects, fold-increase in serum IgG2 antibodies to 3 of the 4 PcPs (4, 6B and 9V) correlated with ICOS^+^ cmT_FH_ cells at D7 (R≥0.46, p≤0.04, S7A Table) while the fold increase in IgG1 antibodies to PcP 9V alone correlated with ICOS^+^ cmT_FH_ cells at D7 (R = 0.45, p = 0.047, S7A Table). In contrast, fold-increase in IgG2 antibodies to none of the 4 PcPs correlated with ICOS^+^ cmT_FH_ cells at D7 in HIV patients (S7A Table). Interestingly, ART-naive HIV patients alone exhibited a correlation of both ICOS^+^ and ICOS^-^ cmT_FH_ cells with the fold-increase in IgG1 antibodies to PcP 4 and 9V (S7A and S7B Table).

## Discussion

We have demonstrated that production of PcP-specific IgG1^+^ and IgG2^+^ ASCs at D7 after vaccination with unconjugated PcPs is associated with the frequency of ICOS^+^ cmT_FH_ cells at that time in HIV seronegative subjects. The fold-increase in serum PcP-specific IgG2 antibodies at D28 also correlated with the frequency of ICOS^+^ cmT_FH_ cells at D7. In contrast, IgG1^+^ and IgG2^+^ ASCs were not associated with the frequency of ICOS^-^ cmT_FH_ cells after vaccination or with the frequency of IgM^+^ or IgG^+^ PcP-specific or total memory B cells before vaccination. We also demonstrated that production of IgG1^+^ and IgG2^+^ ASCs in ART-naive and -treated HIV patients, as well as SPBs in ART-naive patients, were lower after vaccination than in HIV seronegative subjects. Furthermore, IgG1^+^ and IgG2^+^ ASCs were not associated with the frequency of ICOS^+^ cmT_FH_ cells in HIV patients. This observation is likely explained by our finding that ICOS^+^ cmT_FH_ cells did not increase in ART-naive HIV patients, and increased to a lesser and more variable degree than for HIV seronegative subjects, in ART-treated patients (Fig 5C). Given that ICOS^+^ cmT_FH_ cells produced after influenza virus vaccination are capable of inducing memory B cells to differentiate into plasma cells and that their frequency correlates with the amount and affinity of influenza virus-specific antibodies produced [34, 35], our findings suggest that ICOS^+^ cmT_FH_ cells produced after PcP vaccination contribute to the regulation of PcP-specific IgG antibody responses, including isotype diversification, and that this is impaired by HIV-1 infection. Our findings also provide support for previous arguments that lymph node T_FH_ cells and cmT_FH_ cells are dysfunctional in patients with HIV-1 infection [45, 47].

Decreased production of PcP-specific IgG1 and IgG2 antibodies after vaccination in HIV patients might be directly caused by HIV replication, as defects of antibody production and/or isotype diversification have been attributed to the HIV regulatory protein negative factor (nef) and the HIV envelope glycoprotein gp120 [54, 55]. In addition, HIV gp120 may interfere with pneumococcal antibody production by suppressing the variable region heavy gene family 3 (VH3), which is involved in the production of pneumococcal antibodies [56]. However, all of the ART-treated HIV patients in this study had well-controlled HIV infection and had received stable ART for a median time of 9.25 years (interquartile range = 1.3–21.7 years), suggesting that impaired IgG antibody production was not a direct result of HIV-1 replication in these patients. In addition, we have demonstrated that IgG antibody responses to PcPs are not associated with markers of B cell activation or dysfunction in ART-treated HIV patients [57].

Decreased vaccine-induced IgG antibody responses to some antigens in HIV patients have been associated with low nadir CD4^+^ T cell counts [58]. While 46% of HIV patients in our study had nadir CD4^+^ T cell counts of <200 cells/μL, we observed no associations between nadir CD4^+^ T cell count and IgG1^+^ or IgG2^+^ ASCs, though there were weak correlations between nadir CD4^+^ T cell count and fold change in serum IgG2 antibodies to PcP 4 and 9V in ART-treated HIV patients (S7C Table). These findings are in accord with those of previous studies, which have not shown a relationship between nadir CD4^+^ T cell counts and total IgG antibody responses to PcPs in HIV patients, including patients with nadir CD4^+^ T cell counts <200/μL [53].

We suggest that a more likely cause of decreased IgG1 and/or IgG2 antibody responses to PcPs in HIV patients is disruption of lymphoid tissue architecture and GC dysfunction caused by HIV-induced inflammation and fibrosis [59, 60]. This may also be an explanation for our observation of lower frequencies of ICOS^-^ cmT_FH_ cells in ART-treated HIV patients, compared to HIV seronegative subjects (Fig 5B), as ICOS^-^ cmT_FH_ cells likely represent a population of ‘quiescent’ T_FH_ cells produced in GCs [26]. Disruption of lymphoid tissue architecture caused by HIV-induced inflammation and fibrosis is also a likely cause of low proportions of circulating memory B cells in HIV patients [4–6, 61]. In HIV seronegative individuals, memory B cells reacting with PcPs reside in both the IgM^+^ and switched memory B cell subpopulations, though to a different degree depending on age [8, 9]. We therefore examined the association of IgG1^+^ and IgG2^+^ ASCs after vaccination with IgM^+^ and IgG^+^ PcP-specific and total memory B cells prior to vaccination, in addition to ICOS^+^ and ICOS^-^ cmT_FH_ cells after vaccination. In a comprehensive analysis, the parameter, which consistently associated with IgG1^+^ and IgG2^+^ ASCs was ICOS^+^ cmT_FH_ cells. However, while ICOS^+^ cmT_FH_ cells were associated with IgG1^+^ ASCs for all 4 PcP serotypes, this was only observed for serotypes 4 and 6B for IgG2 ASCs. This observation possibly reflects the lower immunogenicity of PcPs 9V and 14 [51] compared with 4 and 6B, as demonstrated here (Fig 4), and the requirement for longer GC reactions to produce IgG2 antibodies [23].

Of interest, in the ART-naive HIV patients alone, the fold-increase in IgG1 antibodies to PcPs 4 and 9V at D28 correlated with proportions of ICOS^-^ as well as ICOS^+^ cmT_FH_ cells at D7 (S7A and S7B Table). Locci et al. [62] demonstrated that ICOS^-^ cmT_FH_ cells (PD-1^+^CXCR3^-^CXCR5^+^CD4^+^) were associated with production of broadly neutralising antibodies to HIV in ART-naive HIV patients, so ‘quiescent’ cmT_FH_ cells may be associated with IgG antibody production in ART-naive HIV patients.

Having demonstrated that expansion of ICOS^+^ cmT_FH_ cells correlated with production of IgG1^+^ and IgG2^+^ ASC at D7 and serum IgG2 antibodies at D28 after vaccination with unconjugated PcPs, we examined the frequency of this cell population prior to vaccination in HIV patients and HIV seronegative subjects. We demonstrated that ART-naive HIV patients had higher proportions of ICOS^+^ but not ICOS^-^ cmT_FH_ when compared with both ART-treated HIV patients and HIV seronegative subjects. Lymph nodes of HIV patients and macaques with SIV infection exhibit increased proportions of GC T_FH_ cells (CXCR5^+^PD1^high^), which correlates with proportions of plasma cells and GC B cells [43–45]. Furthermore, Perreau et al. [44] demonstrated that lymph node T_FH_ cells (CXCR5^+^PD-1^+^Bcl-6^+^) of HIV patients produced IL-21 and supported immunoglobulin production by B cells. Our findings suggest that ICOS^+^ cmT_FH_ cells may be the activated counterpart of these cells in the circulation. In support of this proposal, we have shown that ICOS^+^ cmT_FH_ cells have several characteristics of T_FH_ cells, including IL-21 production, which is lower in HIV patients than HIV seronegative subjects (Abudulai LN et al., manuscript in preparation).

To our knowledge, this is the first study to provide evidence that a memory T_FH_ cell subpopulation (ICOS^+^ cmT_FH_ cells) may regulate isotype switching of B cells to generate an IgG2 antibody response in humans. IgG antibody responses against cell wall polysaccharides of bacteria are enriched for IgG2 antibodies [10, 11, 63], which is likely to reflect structural and functional characteristics of IgG2 that promote an opsonophagocytic IgG antibody response. In addition to those characteristics that may enhance opsonisation of antigens with a high epitope density [12–17], the functional activity of IgG2 antibodies mediated via the hinge and Fc region mainly enhance phagocytosis, though to a lesser degree than IgG3 and IgG1 antibodies. Thus, IgG2 antibodies bind most avidly to the 131H genotype of FcγRIIa [64], which is the major FcγR mediating phagocytosis, and is carried by 75% of individuals and protective against pneumococcal disease [65, 66]. Dysfunction and/or decreased expansion of ICOS^+^ cmT_FH_ cells in HIV patients might therefore compromise the generation of an opsonophagocytic IgG antibody response against PcPs and increase susceptibility to pneumococcal disease [1–3]. However, it should be noted that we have not shown that ICOS^+^ cmT_FH_ cells regulate production of IgG2 antibodies to PcPs *ex vivo* because insufficient ICOS^+^ cmT_FH_ cells could be obtained from the PBMC samples available to undertake cultures with memory B cells stimulated with PcPs and/or mitogens.

Pneumococcal disease remains a significant cause of morbidity and mortality in individuals with HIV-1 infection. While the use of ART and pneumococcal vaccines has reduced the incidence of pneumococcal disease, incidence rates remain higher than in the general population [1, 67]. Both unconjugated and protein-conjugated pneumococcal vaccines are less immunogenic in HIV patients than in HIV negative individuals [68]. Our findings potentially open up new avenues of research for improving the immunogenicity of pneumococcal vaccines in individuals with HIV-1 infection by identifying a cmT_FH_ cell population associated with production of IgG antibodies to PcPs.

Our study had some limitations. We were unable to undertake experiments to determine if isolated ICOS^+^ cmT_FH_ cells enhanced production of IgG1 or IgG2 PcP-specific antibodies by isolated memory B cells in cell cultures. However, the functional effect of ICOS^+^ cmT_FH_ cells has been clearly demonstrated for other antigens [34, 35, 69] and our comprehensive analyses of the relationship between PcP-specific IgG antibody responses and memory B cells or cmT_FH_ cells in HIV seronegative subjects and HIV patients provides compelling evidence that production of PcP-specific IgG antibodies requires expansion of ICOS^+^ cmT_FH_ cells. Also, we did not compare study groups for PcP-specific opsonophagocytic antibodies or maintenance of IgG1 and IgG2 antibodies beyond D28 and determine their relationship with ICOS^+^ cmT_FH_ cells, which should be done in future studies. The number of study subjects vaccinated was limited, especially amongst ART-naive patients, by a protocol requirement to enrol participants who had not received pneumococcal vaccination. However, as we were examining the pathogenesis of HIV-induced disease rather than pneumococcal vaccine efficacy, we believe participant numbers were sufficient. Also, there was a sex imbalance between HIV patients and HIV seronegative subjects but we did not observe differences in pneumococcal vaccine responses related to sex (data not shown).

In summary, we present novel evidence that production of IgG antibodies to PcPs after vaccination is associated with expansion of ICOS^+^ cmT_FH_ cells and that this is impaired by HIV infection and not fully corrected by ART. As production of IgG antibodies, SPBs and ICOS^+^ cmT_FH_ cells reflect GC function, our findings add to the growing body of evidence that GCs are dysfunctional in HIV patients which may contribute to the increased susceptibility to pneumococcal, and possibly meningococcal [70], disease. Additionally, we have identified a cmT_FH_ cell subpopulation that could be investigated in studies of vaccine-induced IgG antibodies to PcPs and/or isotype diversification of IgG antibodies in HIV patients and possibly other individuals with a high risk of pneumococcal disease.

## Materials and methods

### Ethics statement

Informed written consent was obtained from all participants and the study was approved by the Human Research Ethics Committees of Royal Perth Hospital, Perth, Australia (2011/027), the University of Western Australia, Perth, Australia (RA/4/1/4871) and Prince of Wales Hospital, Sydney, Australia (11/020).

### Study participants

HIV patients who were receiving ART (n = 28) or were ART-naive (n = 11) and HIV-negative subjects matched for age (n = 20) were recruited to the study if they had not previously been vaccinated with PcPs. All HIV patients were over the age of 18 and attending clinics in Perth or Sydney. Demographic and clinical data are summarised in S8 Table. All study participants were vaccinated with 0.5mL 23-valent unconjugated PcPs (Pneumovax^™^, Merck, Sharp and Dohme, Whitehouse Station, NJ, USA) in the deltoid muscle of one arm.

### Processing of blood samples

All vaccinated subjects had blood drawn into heparinised tubes before vaccination (D0), and one week (range 7–8 days, D7) and four weeks (range 28–37 days, D28) after vaccination. PBMC were isolated by Ficoll-Hypaque density gradient separation and cryopreserved until analysis. Serum and plasma samples were stored at -80°C. Whole blood CD4^+^ T cell counts were assayed in laboratories accredited by the National Association of Testing Authorities, Australia.

### Enumeration of cmT_FH_ cells, plasmablasts and IgM^+^ and IgG^+^ memory B cells

Circulating memory (cm) T_FH_ cells were enumerated using the following fluorescently-conjugated monoclonal antibodies (mAb): CD3-V450 (clone UCHT1), CD4-V500 (RPA-T4), CD27-PeCy7 (M-T271), CXCR5-Alexa Fluor 488 (RF8B2), ICOS-PE (DX29), PD-1- APC (MIH4) and CD45RA- APC-H7 (HI100) (BD Biosciences, San Jose, CA). Cryopreserved PBMC (2x10^6^ cells) were incubated with mAbs in the dark for 20 minutes, washed and resuspended in phosphate buffered saline (PBS) with 1% bovine serum albumin (BSA). Analyses were performed using a FACS Canto II cytometer (BD Biosciences). A minimum of 200,000 CD4 events were acquired for each sample. Files were exported in FCS 3.0 format and visualised using FlowJo software version 7.6 (Tree Star, Ashland, OR). Characterisation of cmT_FH_ cells was achieved by gating of CD3 and CD4 expression on lymphocytes (side scatter vs. forward scatter) and then sequential gating to identify the proportion of CD4^+^ T cells with a central memory phenotype (CD27^+^CD45RA^-^). Further classification as cmT_FH_ cells was based on co-expression of CXCR5 and PD-1, followed by evaluation of ICOS expression. Expression levels of ICOS and PD-1 in naive T cell populations were utilized to assist with setting gates for expression of these markers on central memory T cells. Proportions of ICOS^+^ and ICOS^-^ cells were determined relative to CD4^+^ T cells (S3 Fig). Our testing protocol for ICOS^+^ cells was validated in two ways. Firstly, in a subset of 13 HIV seronegative subjects, proportions of ICOS^+^ cells in freshly isolated PBMC correlated strongly with proportions of ICOS^+^ cells in cryopreserved PBMC (R = 0.78, p = 0.002, S5A Fig). Secondly, in a subset of 15 HIV patients and HIV seronegative subjects, we confirmed that PD-1 staining using the EH12.2H7 clone (Alexa Fluor 647), which has commonly been used in previous studies, gave comparable results to the PD-1 antibody clone (MIH4) used in our experiments (R = 0.74, p = 0.001, S5B Fig).

For the characterisation of plasmablasts (S2 Fig), the staining panel consisted of the following markers: CD3-V500 (UCHT1, for exclusion), CD20-APC-H7 (2H7), CD27-V450 (M-T271) and CD38-PerCp-Cy5.5 (HIT2) (BD Biosciences). Cryopreserved PBMC (1.5 x 10^6^–2 x 10^6^ cells) were incubated with mAbs in the dark for 20 minutes, washed and resuspended in PBS with 1% BSA. Analysis was done as described above. A minimum of 250,000 lymphocyte events were collected based on the side and forward scatter profile. IgM^+^ memory B cells (CD20^+^CD27^+^IgM^+^IgD^+^) and IgG^+^ switched memory B cells (CD20^+^CD27^+^IgG^+^) were characterised after incubation of PBMC with mAbs to CD3-V500 (UCHT1), CD20-APC-H7 (2H7), CD27-V450 (M-T271), IgM-APC (G20-127) and IgD-PeCy7 (IA6-2) using the protocol described above.

### Assay of serum PcP-specific IgG1 and IgG2 antibodies

IgG1 and IgG2 antibodies to pneumococcal polysaccharide serotypes 4, 6B, 9V and 14 were assayed blindly in serum collected at D0 and D28 after vaccination in ART-treated (n = 28) and ART-naive (n = 11) HIV patients and HIV seronegative subjects (n = 20) using a microsphere-based flow cytometric assay, as previously described [71]. Vaccine responsiveness was assessed by calculating the fold-increase in antibody level between days 0 and 28 for samples with a D0 level <1.3μg/mL [52].

### ELISpot assay for PcP-specific IgG1^+^ and IgG2^+^ ASCs

ELISpot assays were used to enumerate IgG1^+^ and IgG2^+^ ASCs specific for PcP serotypes 4, 6B, 9V and 14 in pre- and post-vaccination cryopreserved PBMC. The four serotypes evaluated elicit strongly immunogenic responses in HIV patients [72, 73]. Briefly, 96-well filter plates (Millipore, San Diego, CA, USA) were coated overnight at 4°C with 10μg/mL purified PcP 4, 9V and 14 and 20μg/mL purified PcP 6B (ATCC, Manassas, VA). Plates were washed and blocked with sterile Roswell Park Memorial Institute Medium (RPMI)-1640 plus 10% BSA for 2 hours at 37°C. Cryopreserved PBMC were thawed using a method to retain ASC viability and function [74]. After assessment of viability by trypan blue exclusion (only cells with a viability of >90% were used), 2 x 10^5^ PBMC were added to the wells of the plate for 18–20 hours at 37°C. The plate was washed with PBS/0.25% Tween (PBST). Antibodies to PcP serotypes 4, 6B, 9V and 14 bound to the plate were detected with alkaline phosphatase-conjugated anti-human IgG1 and IgG2 antibody (MyBiosource, San Diego, CA, USA) for 4 hours at room temperature. Plates were washed three times with PBST and three times with sterilised distilled water and then developed with alkaline phosphatase conjugate substrate kit (Bio-Rad, Hercules, CA, USA). Developed plates were counted using the AID ELISpot reader (AID, Germany). Data are presented as the proportion of IgG1^+^ or IgG2^+^ ASCs per 2x10^5^ PBMC with background values subtracted.

### Enumeration of PcP-specific IgM^+^ and IgG^+^ memory B cells

IgM^+^ and IgG^+^ memory B cells specific for PcP serotypes 4, 6B, 9V and 14 in pre-vaccination cryopreserved PBMC were enumerated by ELISpot assays. PBMC were cultured at 2x10^6^ cells/mL in sterile culture media [RPMI-1640 media plus 10% heat inactivated foetal calf serum (FCS)] supplemented with 1.0μg/mL IL-2 (PeproTech, Rocky Hill, NJ) and 2.5μg/mL R848 (InvivoGen, San Diego, CA) as described previously [75]. Unstimulated cells were cultured in culture media alone. Cells were cultured for 5 days at 37°C, 5% CO_2_. The preparation of ELISpot plates and cell viability was done as described above. The cultured PBMC were washed thoroughly, plated in duplicate onto the ELISpot plates at 5 x10^4^ cells per well and incubated for 16–24 hours at 37°C, 5% CO_2_. Plates were then washed with PBST followed by PBS. Memory B cells specific for PcP 4, 6B, 9V and 14 bound to the plate were detected with alkaline phosphatase-conjugated anti-human IgG and IgM antibody (Invitrogen, Carlsbad, CA) for 4 hours at room temperature. Plates were washed and developed as described above. Data are presented as the proportion of IgG^+^ or IgM^+^ memory B cells per 5x10^4^ PBMC with background values subtracted.

### Statistical analysis

Sample characteristics are summarised using medians, ranges and proportions as appropriate. Differences in baseline characteristics between groups were tested using Mann Whitney test or Fisher’s exact test as appropriate. Differences in IgG1^+^ and IgG2^+^ ASCs between groups at day 7 were assessed using the non-parametric Mann-Whitney test. Correlations between variables were evaluated by the non-parametric Spearman’s rank correlation test. Differences in IgG1^+^ and IgG2^+^ ASC responses between and within groups over time were examined using repeated measures negative binomial regression due to the count nature of the data and the presence of over dispersion. IgG1^+^ and IgG2^+^ ASC proportions were predominantly 0 at D0 and D28 preventing the repeated measures negative binomial analysis from producing a solution. Hence, non-parametric tests (Wilcoxon signed-rank test for within group and Kruskal Wallis for between groups) were performed. To generate p values for plasmablasts, a log transformation was performed and analysed using linear mixed models. To evaluate proportions of ICOS^+^ and ICOS^-^ cmT_FH_ cells, a Wilcoxon signed rank test for within group and Kruskal Wallis for between groups was performed.

The association between PcP-specific IgG1^+^ and IgG2^+^ ASCs after vaccination and a priori selected independent variables, including pre-vaccination CD4^+^ T cell count, proportions of IgM^+^, IgG^+^ PcP-specific and total memory B cells, and post-vaccination ICOS^+^ and ICOS^-^ cmT_FH_ cells, was investigated using a negative binomial regression model. This analysis was performed for each combination of the independent variable and the ASC outcomes. The IRR obtained provided an estimate of the size of the change in the outcome for a one-unit increase in the independent variable. The size of the IRR indicates the strength of the association. Differences between HIV patients and HIV seronegative subjects in the relationship between IgG1^+^ and IgG2^+^ ASCs and the independent variable were tested using an interaction term. p values for the coefficients for each patient group were obtained using model based contrasts. Statistical analyses were performed using Prism Version 5.04 software (GraphPad) and Stata 12 (StataCorp. 2011. Stata Statistical Software: Release 12. College Station, TX: StataCorp LP). For all tests, p<0.05 was considered significant. Adjustments for multiple comparisons were not made due to the exploratory nature of the study. No formal power analyses were performed due to the pilot nature of the study.

## Supporting information

S1 Fig PcP serotype-specific IgG1^+^ and IgG2^+^ ASCs in HIV patients and HIV seronegative subjects pre- and post-vaccination. ASC responses were measured by ELISpot in unstimulated PBMC before (D0), one week (D7) and four weeks (D28) after vaccination with PcPs. Data are presented as ASC/2x10^5^ PBMC with background values subtracted. The horizontal lines indicate median values. (**A**) ART-treated HIV patients (**B**) ART-naive HIV patients (**C**) HIV seronegative subjects to PcP 6B, (**D**) ART-treated HIV patients (**E**) ART-naive HIV patients (**F**) HIV seronegative subjects to PcP 9V and (**G**) ART-treated HIV patients (**H**) ART-naive HIV patients (**I**) HIV seronegative subjects to PcP 14. Repeated measures negative binomial regression analysis and non-parametric tests for IgG1^+^ and IgG2^+^ ASC counts at D0 and D28. n.s., not significant and p<0.05 considered significant. *p value could not be calculated because there was no variance between day 0 and day 28.
(PDF)

S2 Fig Identification of SPB in blood. Representative plot showing the gating strategy to determine the frequency of SPB defined as CD20^-^CD27^++^CD38^++^. Plots shown are from a HIV seronegative subject D0 (top), D7 (middle) and D28 (bottom) post-vaccination.
(PDF)

S3 Fig Identification of cmT_FH_ cells in blood. Representative flow plot, from an ART-treated HIV patient 7 days post-vaccination, showing the gating strategy used to determine the frequency of ICOS^+^ and ICOS^-^ cmT_FH_ cells (CD4^+^CD45RA^-^CXCR5^+^PD-1^+^) as a proportion of total CD4^+^ T cells.
(PDF)

S4 Fig Fold-change in serum IgG1 and IgG2 to PcP serotypes in HIV patients and HIV seronegative subjects at day 28 post-vaccination. (**A**) IgG1 antibody to PcP 4, 6B, 9V and 14 **(B)** IgG2 to PcP 4, 6B, 9V and 14. Data are presented as fold-change in antibody levels between D0 and D28. Differences between groups were tested using Mann-Whitney tests. n.s., not significant and p<0.05 considered significant.
(PDF)

S5 Fig Validation of ICOS and PD-1 expression on ICOS^+^ cmT_FH_ cells. (**A**) Proportions of ICOS^+^ cells in freshly isolated PBMC and cryopreserved PBMC correlate and (**B**) PD-1 staining using mAb clone EH12.2H7 (AF647) and MIH4 (APC) are comparable. Data were analysed by Spearman’s rank correlation test. Linear regression curves are shown for all data points (red line).
(PDF)

S1 Table Associations between the frequency of ICOS^-^ cmT_FH_ cells in HIV seronegative subjects and the IgG1^+^ and IgG2^+^ ASC response to PcPs 4, 6B, 9V and 14. Data are represented as correlation coefficient of % frequency at D7.
(PDF)

S2 Table Associations between the frequency of ICOS^+^ and ICOS^-^ cmT_FH_ cells in ART-treated HIV patients and the IgG1^+^ and IgG2^+^ ASC response to PcPs 4, 6B, 9V and 14. Data represented as correlation coefficient of % frequency at D7.
(PDF)

S3 Table Associations between the frequency of ICOS^+^ and ICOS^-^ cmT_FH_ cells in ART-naive HIV patients and the IgG1^+^ and IgG2^+^ ASC response to PcPs 4, 6B, 9V and 14. Data represented as correlation coefficient of % frequency at D7.
(PDF)

S4 Table Immune correlates of PcP 6B-specific IgG1^+^ and IgG2^+^ ASCs after vaccination with PcPs in HIV patients and HIV seronegative subjects. (PDF)

S5 Table Immune correlates of PcP 9V-specific IgG1^+^ and IgG2^+^ ASCs after vaccination with PcPs in HIV patients and HIV seronegative subjects. (PDF)

S6 Table Immune correlates of PcP 14-specific IgG1^+^ and IgG2^+^ ASCs after vaccination with PcPs in HIV patients and HIV seronegative subjects. (PDF)

S7 Table Correlation between ICOS^+^ cmTFH cells (A) and ICOS- cmTFH cells (B) at D7 and fold-increase in serum levels of PcP-specific IgG1 and IgG2 antibodies at D28. (C) Correlation of fold-increase in serum levels of PcP-specific IgG1 and IgG2 antibodies at D28 with nadir CD4^+^ T cell counts in ART-treated HIV patients. Data were analysed by Spearman’s rank correlation test.
(PDF)

S8 Table Demographic characteristics of study participants. (PDF)
