# Supplementary material for: Production of IgG antibodies to pneumococcal polysaccharides is associated with expansion of ICOS+ circulating memory T follicular-helper cells which is impaired by HIV infection
Source: PLoS One. 2017 May 2;12(5):e0176641. doi: 10.1371/journal.pone.0176641 (PMC5413043; doi:10.1371/journal.pone.0176641)
Supplement: S1 Fig — ASC responses were measured by ELISpot in unstimulated PBMC before (D0), one week (D7) and four weeks (D28) after vaccination with PcPs. Data are presented as ASC/2x105 PBMC with background values subtracted. The horizontal lines indicate median values. (A) ART-treated HIV patients (B) ART-naive HIV patients (C) HIV seronegative subjects to PcP 6B, (D) ART-treated HIV patients (E) ART-naive HIV patients (F) HIV seronegative subjects to PcP 9V and (G) ART-treated HIV patients (H) ART-naive HIV patients (I) HIV seronegative subjects to PcP 14. Repeated measures negative binomial regression analysis and non-parametric tests for IgG1+ and IgG2+ ASC counts at D0 and D28. n.s., not significant and p<0.05 considered significant. *p value could not be calculated because there was no variance between day 0 and day 28. (PDF) [file pone.0176641.s001.pdf]

S1 Figure

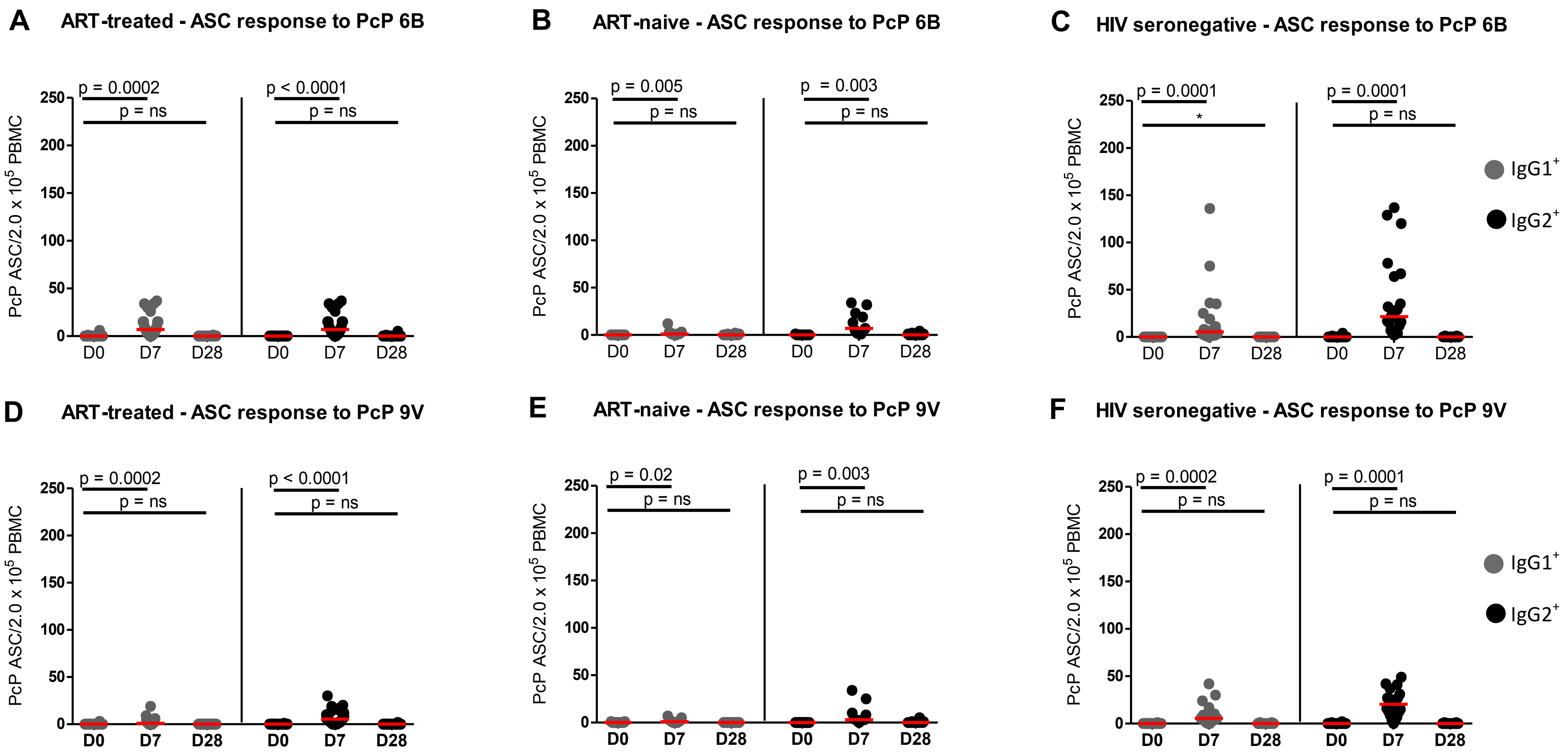
A
ART-treated - ASC response to PcP 6B
B
ART-naive - ASC response to PcP 6B
C
HIV seronegative - ASC response to PcP 6B
D
ART-treated - ASC response to PcP 9V
E
ART-naive - ASC response to PcP 9V
F
HIV seronegative - ASC response to PcP 9V
PcP ASC/2.0 x 10^5 PBMC
p = 0.0002
p < 0.0001
p = 0.005
p = 0.003
p = 0.0001
p = 0.02
p = ns
*
0
50
100
150
200
250
D0
D7
D28
IgG1+
IgG2+

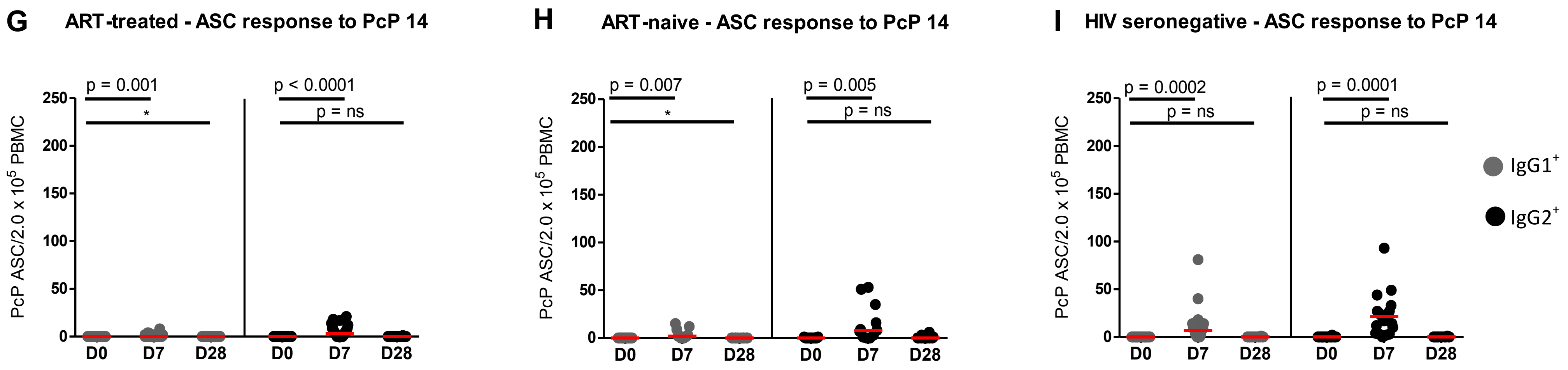
G
ART-treated - ASC response to PcP 14
H
ART-naive - ASC response to PcP 14
I
HIV seronegative - ASC response to PcP 14
PcP ASC/2.0 x 10^5 PBMC
0
50
100
150
200
250
D0
D7
D28
p = 0.001
*
p < 0.0001
p = ns
p = 0.007
p = 0.005
p = 0.0002
p = 0.0001
IgG1+
IgG2+
