# Supplementary figures and images for: Production of IgG antibodies to pneumococcal polysaccharides is associated with expansion of ICOS+ circulating memory T follicular-helper cells which is impaired by HIV infection
Source: PLoS One. 2017 May 2;12(5):e0176641. doi: 10.1371/journal.pone.0176641 (PMC5413043; doi:10.1371/journal.pone.0176641)

**S2 Figure**

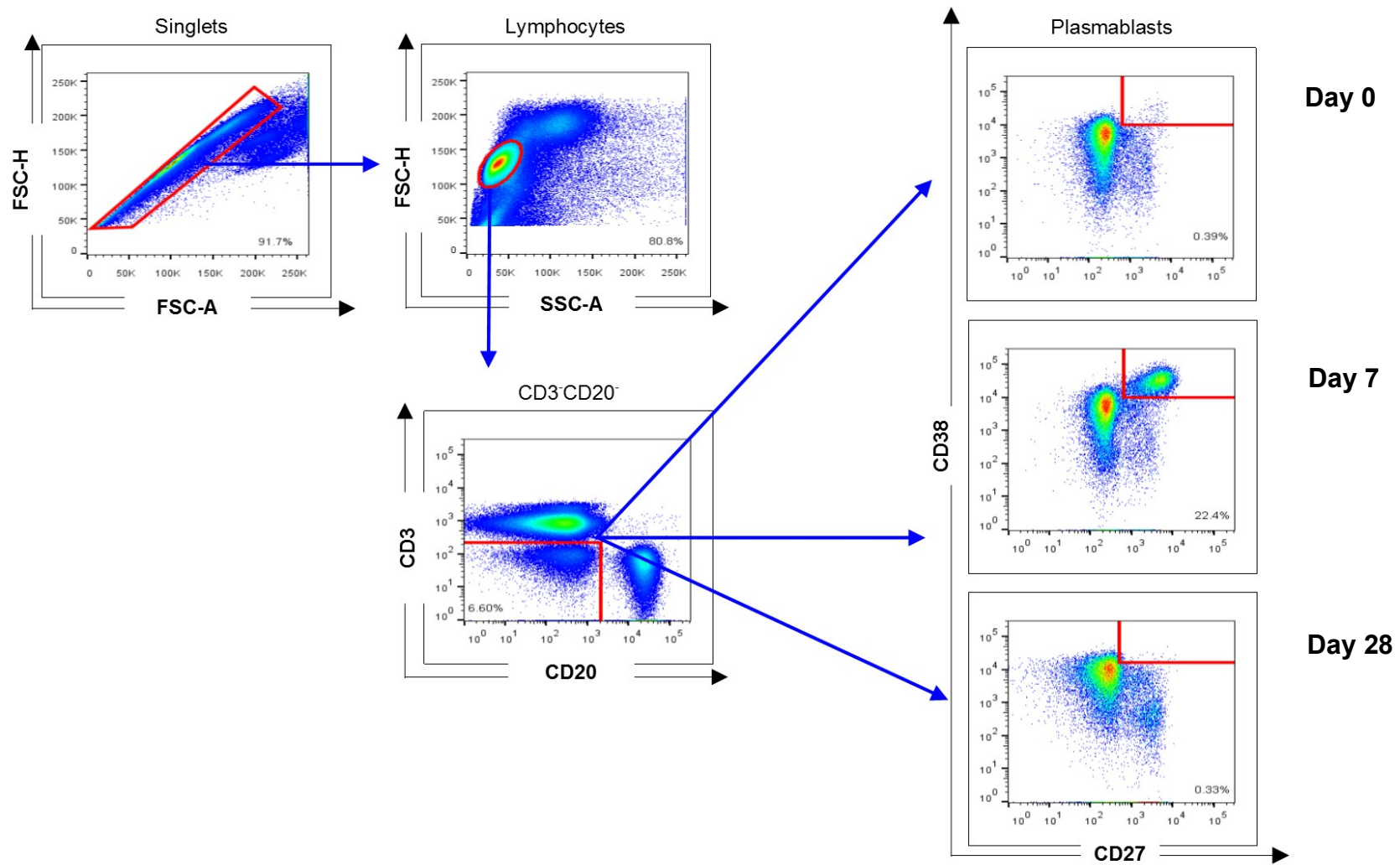

Supplement: S2 Fig — Representative plot showing the gating strategy to determine the frequency of SPB defined as CD20-CD27++CD38++. Plots shown are from a HIV seronegative subject D0 (top), D7 (middle) and D28 (bottom) post-vaccination. (PDF) [file pone.0176641.s002.pdf]

S3 Figure

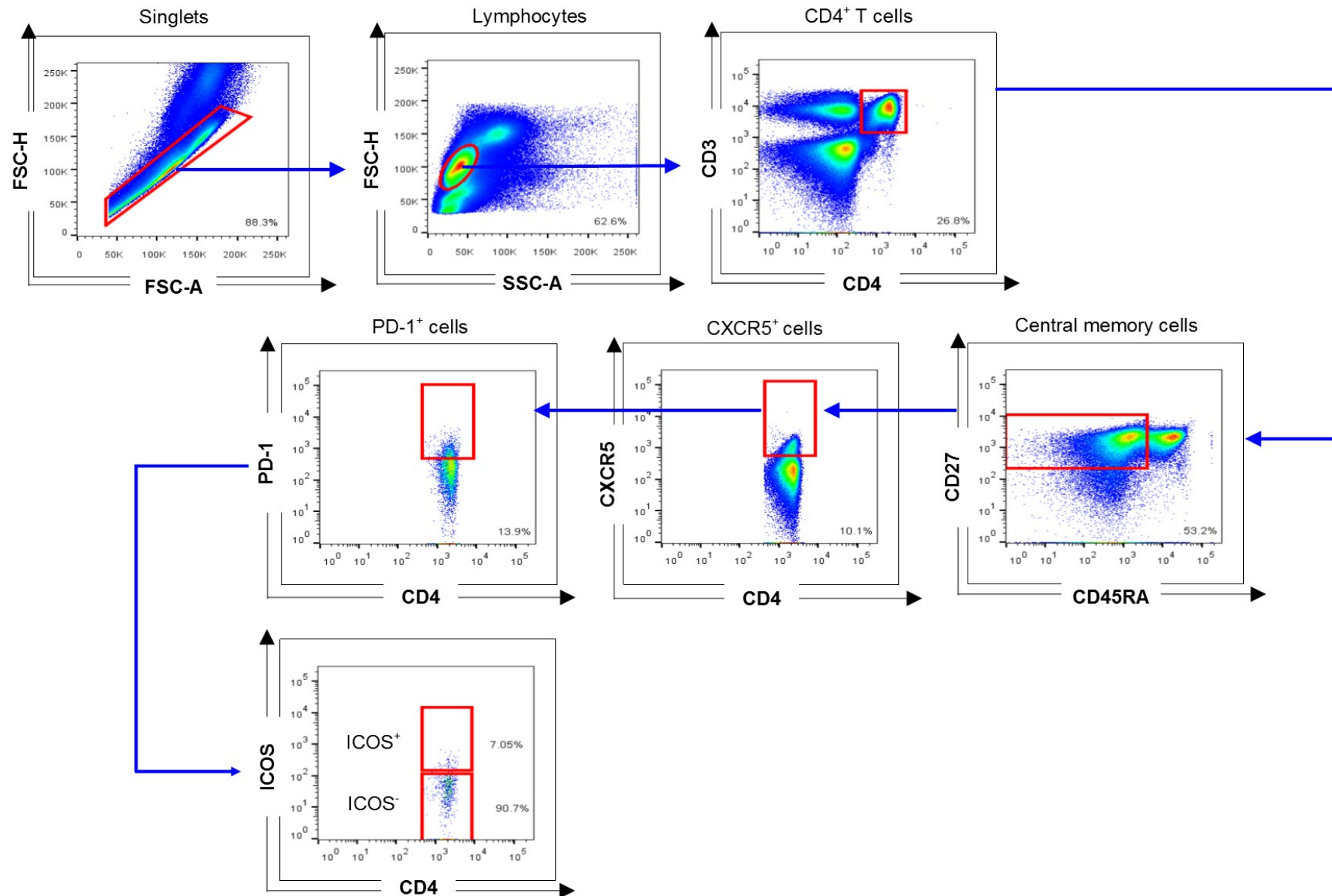

Supplement: S3 Fig — Representative flow plot, from an ART-treated HIV patient 7 days post-vaccination, showing the gating strategy used to determine the frequency of ICOS+ and ICOS- cmTFH cells (CD4+CD45RA-CXCR5+PD-1+) as a proportion of total CD4+ T cells. (PDF) [file pone.0176641.s003.pdf]

**S4 Figure**

- ART-treated
- ART-naïve
- HIV seronegative

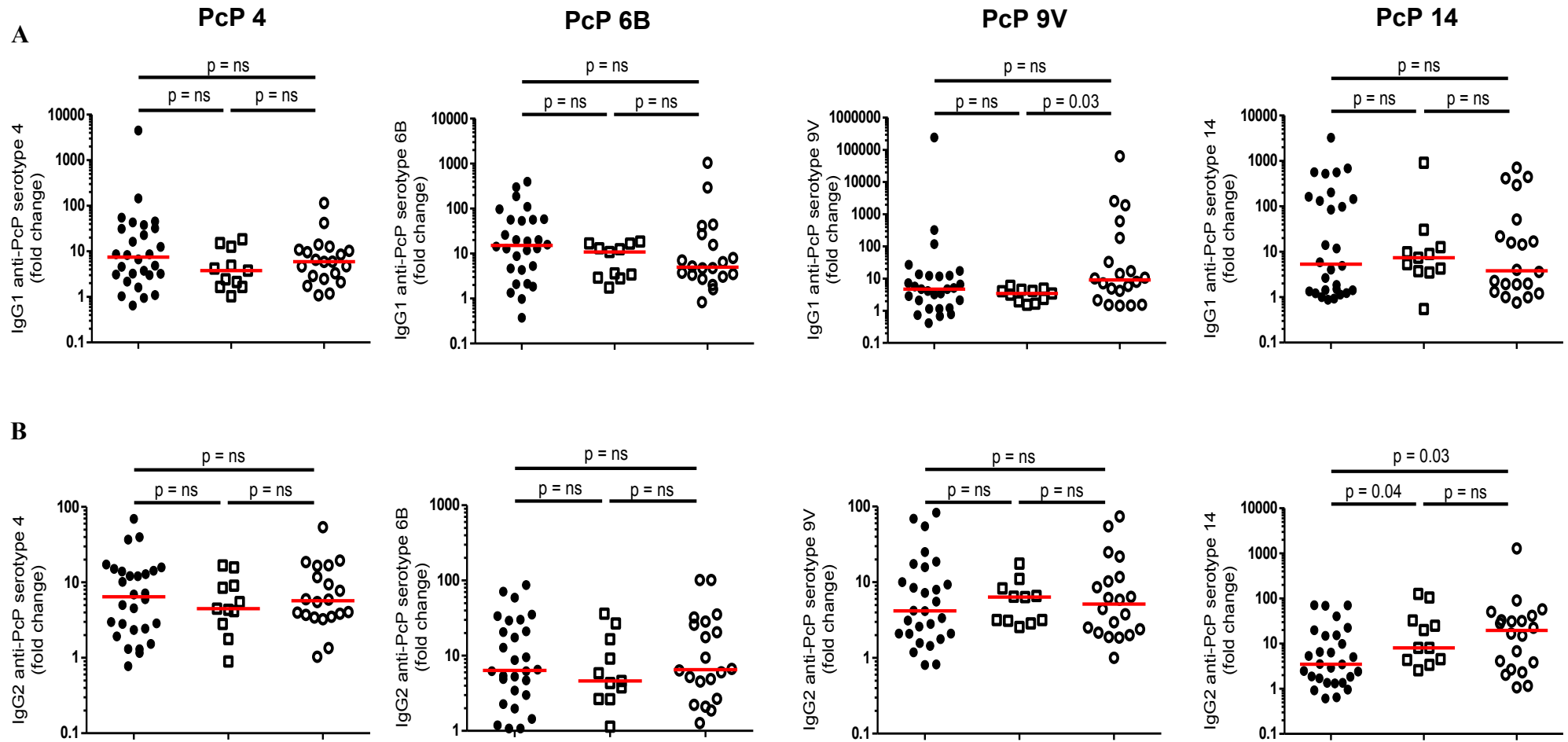

Supplement: S4 Fig — (A) IgG1 antibody to PcP 4, 6B, 9V and 14 (B) IgG2 to PcP 4, 6B, 9V and 14. Data are presented as fold-change in antibody levels between D0 and D28. Differences between groups were tested using Mann-Whitney tests. n.s., not significant and p<0.05 considered significant. (PDF) [file pone.0176641.s004.pdf]

**S5 Figure**

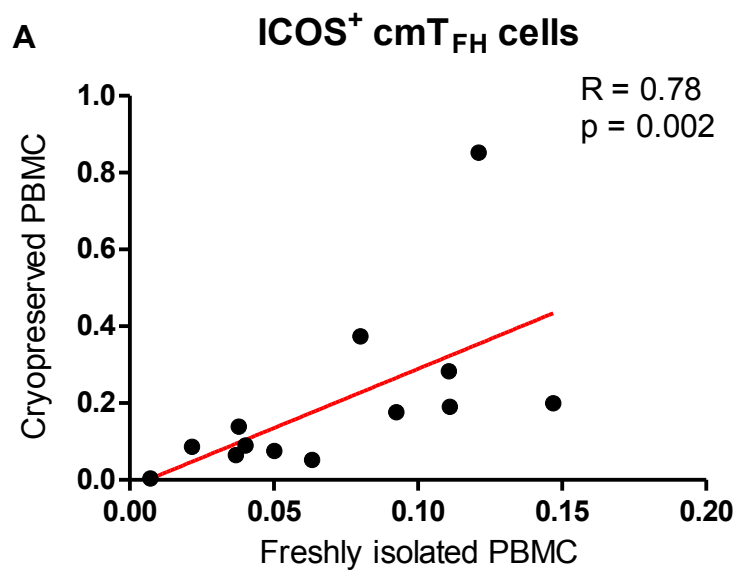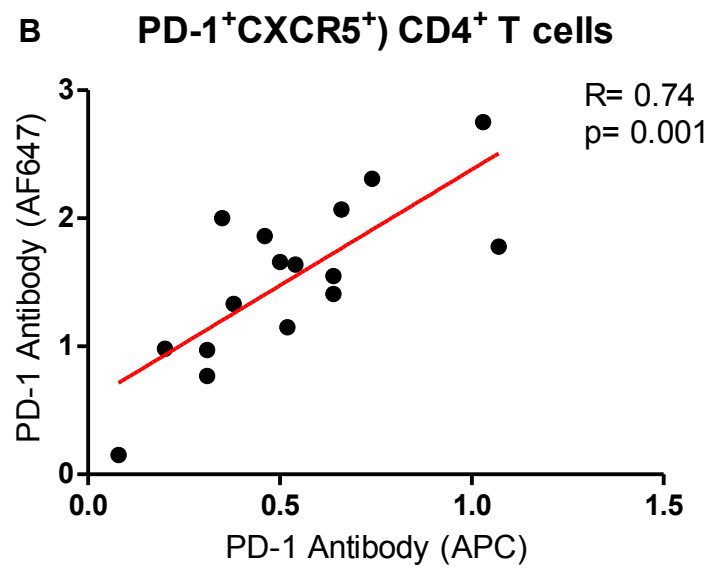

Supplement: S5 Fig — (A) Proportions of ICOS+ cells in freshly isolated PBMC and cryopreserved PBMC correlate and (B) PD-1 staining using mAb clone EH12.2H7 (AF647) and MIH4 (APC) are comparable. Data were analysed by Spearman’s rank correlation test. Linear regression curves are shown for all data points (red line). (PDF) [file pone.0176641.s005.pdf]
