# Supplementary material for: Production of IgG antibodies to pneumococcal polysaccharides is associated with expansion of ICOS+ circulating memory T follicular-helper cells which is impaired by HIV infection
Source: PLoS One. 2017 May 2;12(5):e0176641. doi: 10.1371/journal.pone.0176641 (PMC5413043; doi:10.1371/journal.pone.0176641)
Supplement: S1 Table — Data are represented as correlation coefficient of % frequency at D7. (PDF) [file pone.0176641.s006.pdf]

		PcP 4	PcP 6B	PcP 9V	PcP 14
$IgG1^+$	$ICOS^-$ cmT_{FH}	R = 0.36<br>p = 0.12	R = 0.24<br>p = 0.30	R = 0.02<br>p = 0.92	R = 0.34<br>p = 0.14
$IgG2^+$	$ICOS^-$ cmT_{FH}	R = 0.33<br>p = 0.15	R = 0.33<br>p = 0.16	R = 0.27<br>p = 0.26	R = 0.35<br>p = 0.13
