# Supplementary material for: Production of IgG antibodies to pneumococcal polysaccharides is associated with expansion of ICOS+ circulating memory T follicular-helper cells which is impaired by HIV infection
Source: PLoS One. 2017 May 2;12(5):e0176641. doi: 10.1371/journal.pone.0176641 (PMC5413043; doi:10.1371/journal.pone.0176641)
Supplement: S2 Table — Data represented as correlation coefficient of % frequency at D7. (PDF) [file pone.0176641.s007.pdf]

| | | PcP 4 | PcP 6B | PcP 9V | PcP 14 |
|---|---|---|---|---|---|
| $IgG1^+$ | $ICOS^+$ $cmT_{FH}$ | R = -0.30<br>p = 0.12 | R = -0.01<br>p = 0.95 | R = -0.33<br>p = 0.09 | R = 0.06<br>p = 0.77 |
| | $ICOS^-$ $cmT_{FH}$ | R = -0.17<br>p = 0.37 | R = -0.12<br>p = 0.55 | R = -0.17<br>p = 0.39 | R = 0.11<br>p = 0.58 |
| $IgG2^+$ | $ICOS^+$ $cmT_{FH}$ | R = -0.04<br>p = 0.86 | R = -0.06<br>p = 0.78 | R = -0.01<br>p = 0.94 | R = 0.20<br>p = 0.31 |
| | $ICOS^-$ $cmT_{FH}$ | R = -0.09<br>p = 0.66 | R = -0.08<br>p = 0.70 | R = -0.03<br>p = 0.90 | R = 0.15<br>p = 0.45 |
