# Supplementary material for: Production of IgG antibodies to pneumococcal polysaccharides is associated with expansion of ICOS+ circulating memory T follicular-helper cells which is impaired by HIV infection
Source: PLoS One. 2017 May 2;12(5):e0176641. doi: 10.1371/journal.pone.0176641 (PMC5413043; doi:10.1371/journal.pone.0176641)
Supplement: S3 Table — Data represented as correlation coefficient of % frequency at D7. (PDF) [file pone.0176641.s008.pdf]

		PcP 4	PcP 6B	PcP 9V	PcP 14
$IgG1^{+}$	$ICOS^{+}$ cmT_{FH}	$R = 0.41$<br>$p = 0.21$	$R = 0.27$<br>$p = 0.42$	$R = 0.25$<br>$p = 0.46$	$R = 0.05$<br>$p = 0.89$
	$ICOS^{-}$ cmT_{FH}	$R = 0.35$<br>$p = 0.29$	$R = 0.14$<br>$p = 0.69$	$R = 0.20$<br>$p = 0.56$	$R = 9.3x10^{4}$<br>$p = 0.98$
$IgG2^{+}$	$ICOS^{+}$ cmT_{FH}	$R = 0.36$<br>$p = 0.27$	$R = 0.23$<br>$p = 0.50$	$R = 0.35$<br>$p = 0.29$	$R = 0.23$<br>$p = 0.49$
	$ICOS^{-}$ cmT_{FH}	$R = 0.32$<br>$p = 0.34$	$R = 0.20$<br>$p = 0.56$	$R = 0.32$<br>$p = 0.33$	$R = 0.09$<br>$p = 0.79$
