# Supplementary material for: Production of IgG antibodies to pneumococcal polysaccharides is associated with expansion of ICOS+ circulating memory T follicular-helper cells which is impaired by HIV infection
Source: PLoS One. 2017 May 2;12(5):e0176641. doi: 10.1371/journal.pone.0176641 (PMC5413043; doi:10.1371/journal.pone.0176641)
Supplement: S4 Table — (PDF) [file pone.0176641.s009.pdf]

**Immune correlate**	**Interaction p-value**						**Incident rate ratio (IRR) (95% confidence interval)**					
	ART-treated v HIV seronegative		ART-naive v HIV seronegative		ART-treated v ART-naive		ART-treated HIV patients		ART-naive HIV patients		HIV seronegative subjects	
	$IgG1^+$ ASCs	$IgG2^+$ ASCs	$IgG1^+$ ASCs	$IgG2^+$ ASCs	$IgG1^+$ ASCs	$IgG2^+$ ASCs	$IgG1^+$ ASCs	$IgG2^+$ ASCs	$IgG1^+$ ASCs	$IgG2^+$ ASCs	$IgG1^+$ ASCs	$IgG2^+$ ASCs
$CD4^+$ T cell count (D0), cells/μL	0.23	0.50	0.86	0.85	0.60	0.84	1.00 (0.99,1.00) p = 0.98	0.99 (0.99,1.00) p = 0.39	1.00 (0.99,1.00) p = 0.57	0.99 (0.99,1.00) p = 0.93	1.00 (0.99, 1.00) p = 0.14	1.00 (0.99, 1.00) p = 0.82
Total IgM memory B cells ($CD20^+CD27^+IgM^+$) (D0), %	0.89	0.86	0.28	0.45	0.30	0.42	0.98 (0.87,1.10) p = 0.70	0.98 (0.88,1.08) p = 0.63	0.76 (0.48,1.21) p = 0.24	0.83 (0.58,1.21) p = 0.34	0.99 (0.91, 1.08) p = 0.77	0.97 (0.91, 1.03) p = 0.29
Total IgG memory B cells ($CD20^+CD27^+IgG^+$) (D0), %	0.97	0.36	0.95	0.56	0.98	0.94	0.99 (0.85,1.16) p = 0.96	1.00 (0.89,1.14) p = 0.87	0.99 (0.76,1.29) p = 0.95	0.99 (0.76,1.30) p = 0.99	1.00 (0.80, 1.26) p = 0.99	0.90 (0.74, 1.11) p = 0.33
PcP 6B-specific IgM^+ memory B cells (D0), counts	0.63	0.61	0.99	0.23	0.65	0.65	0.97 (0.90,1.05) p = 0.53	0.99 (0.93,1.07) p = 0.98	0.99 (0.94,1.06) p = 0.93	1.02 (0.97,1.07) p = 0.47	0.99 (0.95, 1.04) p = 0.88	0.98 (0.94,1.02) p = 0.29
PcP 6B-specific IgG^+ memory B cells (D0), counts	0.60	0.77	0.27	0.07	0.19	0.29	0.95 (0.86,1.04) p = 0.95	0.97 (0.90,1.06) p = 0.52	1.03 (0.94,1.13) p = 0.46	1.03 (0.97,1.09) p = 0.37	0.98 (0.93,1.05) p = 0.33	0.96 (0.92,1.00) p = 0.07
$ICOS^+$ cmT_{FH} cells (D7), %	**<0.001**	**<0.001**	**<0.001**	**<0.001**	0.77	0.52	0.29 ($1.84x10^{-3}$, 44.28) p = 0.63	0.09 ($1.26x10^{-3}$, 6.06) p = 0.26	0.64 (0.09, 4.19) p = 0.64	0.40 (0.06, 2.69) p = 0.35	$4.47x10^{10}$ ($4.9x10^{5}$, $4.12x10^{15}$) **p = <0.001**	$3.25x10^{8}$ ($8.53x10^{3}$, $1.24x10^{13}$) **p = <0.001**
$ICOS^-$ cmT_{FH} cells (D7), %	0.44	0.43	0.95	0.84	0.42	0.39	0.07 ($9.29x10^{-5}$, 49.81) p = 0.42	0.18 ($6.73x10^{-4}$, 45.60) p = 0.54	1.15 (0.17, 7.92) p = 0.89	2.17 (0.44, 10.67) p = 0.34	1.05 (0.15,7.56) p = 0.96	1.75 (0.48, 6.42) p = 0.40
