# Supplementary material for: Production of IgG antibodies to pneumococcal polysaccharides is associated with expansion of ICOS+ circulating memory T follicular-helper cells which is impaired by HIV infection
Source: PLoS One. 2017 May 2;12(5):e0176641. doi: 10.1371/journal.pone.0176641 (PMC5413043; doi:10.1371/journal.pone.0176641)
Supplement: S5 Table — (PDF) [file pone.0176641.s010.pdf]

**Immune correlate**	**Interaction p-value**						**Incident rate ratio (IRR) (95% confidence interval)**					
	ART-treated v HIV seronegative		ART-naive v HIV seronegative		ART-treated v ART-naive		ART-treated HIV patients		ART-naive HIV patients		HIV seronegative subjects	
	$IgG1^+$ ASCs	$IgG2^+$ ASCs	$IgG1^+$ ASCs	$IgG2^+$ ASCs	$IgG1^+$ ASCs	$IgG2^+$ ASCs	$IgG1^+$ ASCs	$IgG2^+$ ASCs	$IgG1^+$ ASCs	$IgG2^+$ ASCs	$IgG1^+$ ASCs	$IgG2^+$ ASCs
$CD4^+$ T cell count (D0), cells/μL	0.45	0.53	0.79	0.80	0.47	0.90	1.00 (0.99,1.00) p = 0.33	0.99 (0.99,1.00) p = 0.38	0.99 (0.99,1.00) p = 0.67	0.99 (0.99,1.00) p = 0.83	0.99 (0.99, 1.00) p = 0.81	1.00 (0.99, 1.00) p = 0.89
Total IgM memory B cells ($CD20^+CD27^+IgM^+$) (D0), %	0.99	0.74	0.08	**0.045**	0.08	0.06	1.00 (0.91,1.10) p = 0.97	0.99 (0.91,1.08) p = 0.86	0.61 (0.35,1.06) p = 0.08	0.73 (0.53,0.99) **p = 0.048**	1.00 (0.93, 1.08) p = 0.98	1.00 (0.96, 1.06) p = 0.72
Total IgG memory B cells ($CD20^+CD27^+IgG^+$) (D0), %	0.86	0.53	0.81	0.95	0.92	0.60	0.92 (0.75,1.14) p = 0.46	1.04 (0.92,1.17) p = 0.54	0.94 (0.69,1.28) p = 0.71	0.97 (0.77,1.23) p = 0.79	0.90 (0.73, 1.11) p = 0.33	0.96 (0.77, 1.19) p = 0.71
PcP 9V-specific IgM^+ memory B cells (D0), counts	0.06	0.32	0.09	**0.03**	0.90	**0.007**	1.02 (0.99,1.05) p = 0.26	0.94 (0.89,0.99) **p = 0.01**	1.02 (0.98,1.06) p = 0.34	1.01 (0.99,1.05) p = 0.26	0.98 (0.95,1.01) p = 0.13	0.97 (0.94,1.00) p = 0.06
PcP 9V-specific IgG^+ memory B cells (D0), counts	0.06	0.40	0.16	**0.04**	0.97	**0.02**	1.02 (0.99,1.06) p = 0.18	0.95 (0.89,1.00) p = 0.06	1.02 (0.97,1.08) p = 0.39	1.03 (0.99,1.08) p = 0.18	0.98 (0.95,1.01) p = 0.19	0.98 (0.95,1.00) p = 0.10
$ICOS^+$ cmT_{FH} cells (D7), %	**<0.001**	0.09	**0.001**	0.15	0.08	0.40	$1.5x10^{-3}$ ($2.82x10^{-6}$, 0.83) **p = 0.04**	0.89 ($1.61x10^{-3}$, 8.68) p = 0.33	0.66 (0.07, 5.93) p = 0.71	0.12 (0.13, 6.24) p = 0.90	$3.47x10^{7}$ ($1.13x10^{3}$, $1.07x10^{12}$) **p = 0.001**	$5.7x10^{3}$ (0.04, $7.60x10^{8}$) p = 0.15
$ICOS^-$ cmT_{FH} cells (D7), %	0.05	0.29	0.65	0.62	**0.04**	0.23	$9.5x10^{-4}$ ($7.78x10^{-7}$, 1.16) p = 0.06	0.14 ($1.12x10^{-3}$, 17.93) p = 0.43	2.13 (0.42, 10.89) p = 0.36	3.17 (0.77, 12.97) p = 0.11	1.32 (0.36, 4.80) p = 0.68	2.04 (0.71, 5.86) p = 0.19
