# Supplementary material for: Production of IgG antibodies to pneumococcal polysaccharides is associated with expansion of ICOS+ circulating memory T follicular-helper cells which is impaired by HIV infection
Source: PLoS One. 2017 May 2;12(5):e0176641. doi: 10.1371/journal.pone.0176641 (PMC5413043; doi:10.1371/journal.pone.0176641)
Supplement: S6 Table — (PDF) [file pone.0176641.s011.pdf]

**Immune correlate**	**Interaction p-value**						**Incident rate ratio (IRR) (95% confidence interval)**					
	ART-treated v HIV seronegative		ART-naive v HIV seronegative		ART-treated v ART-naive		ART-treated HIV patients		ART-naive HIV patients		HIV seronegative subjects	
	$IgG1^+$ ASCs	$IgG2^+$ ASCs	$IgG1^+$ ASCs	$IgG2^+$ ASCs	$IgG1^+$ ASCs	$IgG2^+$ ASCs	$IgG1^+$ ASCs	$IgG2^+$ ASCs	$IgG1^+$ ASCs	$IgG2^+$ ASCs	$IgG1^+$ ASCs	$IgG2^+$ ASCs
$CD4^+$ T cell count (D0), cells/μL	0.50	0.31	0.79	0.96	0.92	0.56	1.00 (0.99,1.00) p = 0.71	0.99 (0.99,1.00) p = 0.09	1.00 (0.99,1.00) p = 0.80	1.00 (0.99,1.00) p = 0.93	1.00 (0.99, 1.00) p = 0.20	1.00 (0.99, 1.00) p = 0.95
Total IgM memory B cells ($CD20^+CD27^+IgM^+$) (D0), %	0.99	0.12	0.14	**0.02**	0.15	0.09	0.91 (0.79,1.05) p = 0.21	0.89 (0.78,0.99) p = 0.05	0.64 (0.41, .01) p = 0.06	0.63 (0.44,0.91) **p = 0.02**	0.91 (0.82, 1.01) p = 0.07	0.99 (0.93, 1.05) p = 0.66
Total IgG memory B cells ($CD20^+CD27^+IgG^+$) (D0), %	**0.04**	0.65	0.11	0.35	0.57	0.13	0.62 (0.40,0.97) **p = 0.04**	1.07 (0.93,1.24) p = 0.32	0.74 (0.51,1.07) p = 0.11	0.84 (0.64,1.11) p = 0.23	1.07 (0.82, 1.39) p = 0.64	1.00 (0.79, 1.29) p = 0.97
PcP 14-specific IgM^+ memory B cells (D0), counts	0.41	0.54	0.91	0.42	0.33	0.92	1.04 (0.94,1.15) p = 0.42	0.99 (0.92,1.08) p = 0.92	0.98 (0.93,1.04) p = 0.57	0.99 (0.95,1.04) p = 0.79	0.99 (0.92, 1.07) p = 0.77	0.97 (0.92,1.02) p = 0.21
PcP 14-specific IgG^+ memory B cells (D0), counts	0.59	0.86	0.60	0.81	0.40	0.99	1.04 (0.92,1.19) p = 0.51	0.99 (0.92,1.08) p = 0.90	0.98 (0.92,1.05) p = 0.56	0.99 (0.95,1.05) p = 0.85	1.00 (0.95,1.07) p = 0.89	0.99 (0.94, 1.03) p = 0.56
$ICOS^+$ cmT_{FH} cells (D7), %	**0.005**	0.09	**0.001**	**0.045**	0.63	0.64	1.02 ($2.76x10^{-3}$, 376.57) p = 0.99	2.63 (0.02, 409.06) p = 0.71	0.20 (0.01, 3.56) p = 0.27	0.73 (0.11, 4.70) p = 0.74	$3.86x10^9$ ($2.8x10^3$, $5.30x10^{15}$) **p = 0.002**	$2.3x10^5$ (1.09, $4.81x10^{10}$) **p = 0.048**
$ICOS^-$ cmT_{FH} cells (D7), %	0.84	0.88	0.37	0.37	0.89	0.90	1.13 ($5.00x10^{-4}$, 2557.05) p = 0.97	1.36 ($1.76x10^{-3}$, 1044.39) p = 0.93	0.64 (0.07, 5.65) p = 0.69	0.88 (0.19, 4.08) p = 0.87	2.54 (0.33, 19.24) p = 0.37	2.31 (0.55, 9.67) p = 0.25
