# Supplementary material for: Production of IgG antibodies to pneumococcal polysaccharides is associated with expansion of ICOS+ circulating memory T follicular-helper cells which is impaired by HIV infection
Source: PLoS One. 2017 May 2;12(5):e0176641. doi: 10.1371/journal.pone.0176641 (PMC5413043; doi:10.1371/journal.pone.0176641)
Supplement: S7 Table — Data were analysed by Spearman’s rank correlation test. (PDF) [file pone.0176641.s012.pdf]

A.

	Fold-increase in IgG1 PcP-specific antibodies at D28				Fold-increase in IgG2 PcP-specific antibodies at D28			
	PcP 4	PcP 6B	PcP 9V	PcP 14	PcP 4	PcP 6B	PcP 9V	PcP 14
HIV seronegative subjects (n=20)	R=0.36 p=0.12	R= -0.09 p=0.70	**R=0.45 p=0.047**	R=0.25 p=0.28	**R=0.50 p=0.02**	**R=0.54 p=0.01**	**R=0.46 p=0.04**	R=0.35 p=0.13
ART-naive HIV patients (n=11)	**R=0.60** p=0.05	R= -0.04 p=0.92	**R=0.67 p=0.02**	R=0.24 p=0.48	R=0.25 p=0.47	R= -0.28 p=0.40	R=0.23 p=0.50	R=0.09 p=0.79
ART-treated HIV patients (n=28)	R= -0.20 p=0.30	R= -0.07 p=0.74	R= -0.28 p=0.15	***R= -0.63 p=0.0003***	R= -0.19 p=0.34	***R= -0.41 p=0.03***	R= -0.15 p=0.44	R= -0.29 p=0.14

B.

	Fold-increase in IgG1 PcP-specific antibodies at D28				Fold-increase in IgG2 PcP-specific antibodies at D28			
	PcP 4	PcP 6B	PcP 9V	PcP 14	PcP 4	PcP 6B	PcP 9V	PcP 14
HIV seronegative subjects (n=20)	R=0.31 p=0.19	R= -0.14 p=0.55	R=0.08 p=0.73	R=0.03 p=0.89	**R=0.56 p=0.01**	R= -0.06 p=0.81	R=0.20 p=0.41	R= -0.02 p=0.94
ART-naive HIV patients (n=11)	**R=0.71 p=0.01**	R= -0.05 p=0.89	**R=0.65 p=0.03**	R=0.34 p=0.31	R=0.31 p=0.36	R= -0.25 p=0.45	R=0.07 p=0.83	R=$9x10^{-2}$ p=0.98
ART-treated HIV patients (n=28)	R= -0.21 p=0.29	R= -0.13 p=0.51	R= -0.04 p=0.84	***R= -0.46 p=0.01***	R= -0.20 p=0.31	R= -0.07 p=0.72	R=0.07 p=0.83	R= -0.33 p=0.08

Correlations with an R value >0.4 and p values <0.05 are bolded while negative correlations are also italicized.
