# Supplementary material for: Production of IgG antibodies to pneumococcal polysaccharides is associated with expansion of ICOS+ circulating memory T follicular-helper cells which is impaired by HIV infection
Source: PLoS One. 2017 May 2;12(5):e0176641. doi: 10.1371/journal.pone.0176641 (PMC5413043; doi:10.1371/journal.pone.0176641)
Supplement: S8 Table — (PDF) [file pone.0176641.s013.pdf]

**Characteristic**	**ART-treated HIV patients**	**ART-naive HIV patients**	**HIV seronegative subjects**
**No. Subjects**	28	11	20
**Age (years)**	50.5[a] (30 – 72)	37 (19 – 48)	52.5 (28 – 76)
**Sex, male/female**	26/2[b]	8/3	12/8
**$CD4^+$ T cell count (cells/µL)**	774[c] (310 – 2058)	646 (406 – 1120)	985 (580 – 1500)
**$CD8^+$ T cell count (cells/µL)**	945[d] (308 – 1836)	1012 (532 – 1972)	493 (162 – 810)
**Nadir $CD4^+$ T cell count (cells/µL)**	233[e] (16 – 624)	561 (225 – 1120)	N/A
**B cells (% of lymphocytes)**	10[f] (4 – 22)	9 (4 -23)	15 (6 – 25)
**HIV-1 viral load (copies/mL)**	<40	6310[g] (<40-288403)	N/A
**Years on ART**	9.25 (1.3 - 21.7)	N/A	N/A

[a] Data presented as median (range)

[b] Gender significantly different between ART-treated and HIV seronegative subjects (p = *0.01*; Fisher's exact test).

[c] $CD4^+$ T cell counts significantly different between ART-treated patients ($p = 0.007$) and ART-naive ($p = 0.001$) compared to HIV seronegative subjects (Mann-Whitney t-test).

[d] $CD8^+$ T cell counts significantly different between ART-treated patients ($p = 0.004$) and ART-naive ($p = 0.008$) compared to HIV seronegative subjects (Mann-Whitney t-test).

[e] Nadir $CD4^+$ T cell count significantly different between ART-treated and ART-naive HIV patients ($p = 0.0007$; Mann-Whitney t-test).

[f] B cells significantly different between ART-treated patients and HIV seronegative subjects ($p = 0.01$; Mann-Whitney t-test).

[g] ART-naive group included two patients with an elite controller phenotype
